# WiNGPT-32B: An Open-Source, Locally Deployable LLM for RECIST Assessment via Chained Task Execution Using Radiology Report Text

**DOI:** 10.3390/diagnostics16132020

**Published:** 2026-06-28

**Authors:** Lingyun Wang, Lu Zhang, Yaping Zhang, Lin Zhang, Xueqian Xie

**Affiliations:** Radiology Department, Shanghai General Hospital, Shanghai Jiao Tong University School of Medicine, Haining Rd. 100, Shanghai 200080, China; wanglingyun0514@163.com (L.W.); zhangluuuu@hotmail.com (L.Z.); zhangyaping.ok@163.com (Y.Z.); linzhang2753@163.com (L.Z.)

**Keywords:** Response Evaluation Criteria in Solid Tumors (RECIST), radiology reports, medical reasoning, large language model

## Abstract

**Objective**: The objective of this study was to construct a large language model (LLM) for the Response Evaluation Criteria in Solid Tumors (RECIST) assessment using exclusively longitudinal radiology report text. **Methods**: This study included 258 patients with solid tumors, encompassing 2065 longitudinal CT/MRI examination time points. We developed WiNGPT-32B, an open-source and locally deployable LLM, by infusing it with domain-specific medical knowledge and optimizing it via knowledge distillation, using GPT-4 as the teacher model. Central to its architecture is the Chained Task Execution (CTE) framework, which structures RECIST assessment into four modular components: lesion diameter extraction, sum of longest diameter computation, tumor response classification, and report generation. Model performance (accuracy, recall, precision, and F1 score) was benchmarked against GPT-4 and a single radiologist, utilizing the consensus of three independent radiologists as the reference standard. **Results**: The number of patients with imaging time points was 212 (82.2%) with 4–10, 36 (13.9%) with 11–20, and 10 (3.9%) with >20 time points. For target lesions, the successful extraction rate of WiNGPT-32B was 0.934 (95% CI: 0.922–0.944), which was slightly higher than that of GPT-4 0.920 (0.907–0.931; *p* = 0.083). In five-category RECIST classification (complete response, partial response, stable disease, progressive disease, and not evaluable), WiNGPT-32B achieved an overall accuracy of 0.805 (0.786–0.823), significantly higher than GPT-4 (0.699, 0.678–0.720; *p* < 0.001) but lower than the radiologist (0.915, 0.901–0.928; *p* < 0.001). For progressive disease, WiNGPT-32B had an F1 score of 0.841 (0.813–0.870), significantly outperforming GPT-4’s 0.755 (0.720–0.790), and approaching the radiologist’s 0.922 (0.902–0.942). **Conclusions**: WiNGPT-32B demonstrates the feasibility of a text-only, open-source LLM with the CTE framework for longitudinal RECIST assessment, with promising performance in detecting disease progression.

## 1. Introduction

Assessing tumor response to anticancer therapies, such as chemotherapy or targeted therapy is a cornerstone of clinical oncology, directly guiding treatment adjustment [1,2]. Solid tumors account for approximately 90% of adult malignancies [3], and the Response Evaluation Criteria in Solid Tumors (RECIST) serves as the standard framework for assessing therapeutic response by providing a standardized approach to lesion size measurement and tumor burden quantification [4,5].

However, RECIST-based evaluation involves complex, multi-step processes, including lesion extraction, the sum of lesion diameters (SLD) calculation, temporal comparison, new lesion detection, and response classification. These steps are often not fully documented in routine radiology reports. In busy clinical settings, this process imposes a substantial cognitive burden on radiologists, with inter-rater variability and delayed treatment decision-making. Incorporating RECIST results into routine radiology reports would improve the formulation of treatment plans.

LLMs are relevant to this setting because they can process unstructured longitudinal report text, extract lesion measurements, compare findings across time points, and generate structured RECIST-based outputs when appropriately constrained. Current commercial tools (e.g., syngo.via MM Oncology) assist with lesion measurement but rely on imaging data, making them inaccessible in scenarios where only radiology reports are available, e.g., resource-limited hospitals or retrospective cohort analyses. Meanwhile, large language models (LLMs) have shown promise in medical text processing [6,7,8,9], but their utility in RECIST evaluation is constrained by two critical limitations. First, most radiology-focused LLMs prioritize single-turn tasks, such as report generation or impression simplification [10], and lack multi-step reasoning capabilities, essential for integrating longitudinal data and performing SLD calculations, which are core to RECIST. For instance, Zhang et al. constructed an LLM to generate radiological impressions from imaging descriptions [11], and Doshi et al. applied four LLMs to simplify radiological impressions [8]. Second, State-of-the-Art LLMs are either closed-source (e.g., GPT-4) [12] or overly large (>100 B parameters) [13], hindering local deployment, increasing computational costs, and raising patient data privacy concerns, a key ethical issue in medical artificial intelligence. Even advanced open-source LLMs, e.g., DeepSeek-R1, achieve only 74% accuracy in RECIST evaluation, highlighting the need for tailored multi-step reasoning frameworks [14].

To address these gaps, we constructed a 32-billion-parameter LLM (WiNGPT-32B) with two key innovations: (1) a chained task execution (CTE) framework that decomposes complex RECIST evaluation into modular, sequential subtasks (lesion extraction, SLD computation, temporal comparison, new lesion detection, and response classification) to enable structured multi-step reasoning; (2) knowledge distillation to imbue RECIST-specific clinical reasoning into a compact student model.

This study aimed to verify whether WiNGPT-32B can achieve RECIST evaluation accuracy comparable to experienced radiologists, and to demonstrate its superiority over other closed-source LLMs, such as GPT-4, in multi-step clinical reasoning.

## 2. Materials and Methods

### 2.1. Study Sample

This retrospective study was approved by the Institutional Review Board, with informed consent waived. Patients with solid tumors who underwent CT and/or MRI examinations for treatment response evaluation were retrospectively included from January 2015 to December 2024 at two academic hospitals.

Inclusion criteria were (1) pathologically confirmed solid tumors (e.g., lung, pancreatic, and liver cancer) with CT and/or MRI for therapy response monitoring; (2) ≥4 imaging time points per patient to enable longitudinal SLD comparison and response classification (consistent with RECIST 1.1’s recommended therapy monitoring interval of 6–8 weeks) [5]; (3) radiology reports with clear descriptions of ≥1 measurable target lesion (per RECIST 1.1: tumor long diameter > 10 mm or lymph node short axis > 15 mm); and (4) complete clinical treatment history. Exclusion criteria were (1) suboptimal image quality in ≥1 examination; (2) comorbidities interfering with lesion visualization, e.g., severe lung infection and massive ascites; and (3) radiology reports with ambiguous lesion descriptions, e.g., mildly enlarged lymph nodes, without size quantification.

A single time point could encompass multiple imaging evaluations of different body parts (e.g., chest CT + abdominal MRI) within 14 days. For such cases, all corresponding radiology reports were integrated to extract comprehensive lesion information (target/non-target lesions and lymph nodes), in line with the requirement of RECIST 1.1 for whole-body lesion tracking [5].

### 2.2. LLM Construction

An LLM (WiNGPT-32B) was developed based on the open-source Qwen2.5-32B model as the backbone, which adopts a transformer architecture [15], enhanced by three key components to optimize text processing:

Rotary Positional Embedding (RoPE) captures long-range dependencies between longitudinal radiology reports by rotating query/key matrices with angle-based transformations, and it is critical for temporal lesion comparison [16].

Root Mean Square Layer Normalization (RMSNorm) stabilizes the training of large-scale medical text data by normalizing input features via root mean square calculations, reducing gradient instability [17].

Swish–Gated Linear Unit (SwiGLU) is a hybrid activation function that balances model expressiveness and computational efficiency, and is essential for processing complex lesion descriptions [18].

The pre-training dataset included five types of medical text (Table S1), totaling 247,500 instances: (1) auxiliary diagnostic data (6000 instances, including tumor imaging diagnostic reasoning); (2) quality-control extraction data (2000 instances, based on electronic medical record quality control rules); (3) examination report generation data (180,000 instances, covering CT/MRI); (4) medical question-and-answer data (56,000 instances, including RECIST-related questions); and (5) information extraction data (3500 instances). All datasets were de-identified to comply with the Health Insurance Portability and Accountability Act (HIPAA) and local ethics requirements.

**Figure 1:**
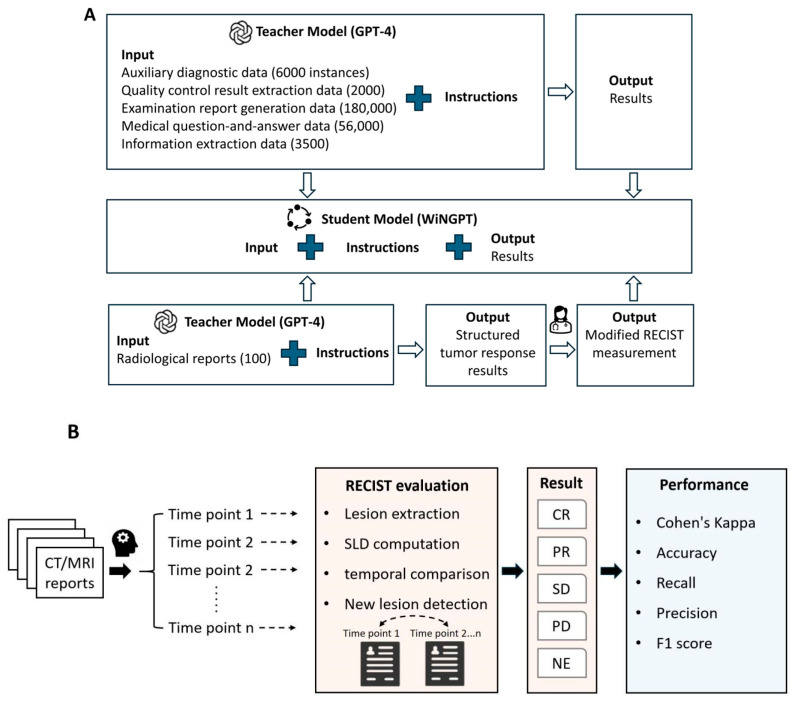
LLM-based RECIST tumor response assessment: Training process (teacher–student model) and evaluation framework. (**A**) Training process: GPT-4 was used as the teacher model to generate structured outputs from medical text data and RECIST-related instructions. The student model, WiNGPT-32B, was trained using general medical text data and GPT-4-generated structured RECIST outputs derived from 100 radiology reports after expert modification. (**B**) Evaluation framework: Longitudinal CT/MRI reports from multiple time points were used as input. The model performed RECIST evaluation through lesion extraction, SLD computation, temporal comparison, and new lesion detection, followed by classification into CR, PR, SD, PD, or NE. Model performance was assessed using Cohen’s kappa, accuracy, recall, precision, and F1 score. Abbreviations: LLM = large language model; SLD = sum of length diameters; CR = complete response; PR = partial response; SD = stable disease; PD = progressive disease; NE = not evaluable.

To imbue clinical reasoning, a knowledge-distillation process was implemented using GPT-4 (teacher model) and WiNGPT-32B (student model) (Figure 1) [19,20]. First, 100 training queries were randomly selected from the pre-training dataset. GPT-4 generated RECIST-compliant responses via a structured prompt template, e.g., “Extract target lesions from the report and calculate SLD per RECIST 1.1.”. Second, two experts validated the accuracy and completeness of the GPT-4 outputs. Finally, WiNGPT-32B was fine-tuned via autoregressive language modeling on the distilled dataset, with cross-entropy loss minimized between the student’s token distribution and the teacher’s responses.

Training was conducted on a Linux platform (Ubuntu 20.04, Canonical Ltd., London, UK) with 8 graphics processing units (A800-80GB, Nvidia, Santa Clara, CA, USA), with a total of 640 GB random-access memory. Model inference for local deployment was performed on 2 GPUs (Titan RTX, Nvidia) with 48 GB memory.

### 2.3. Chained Task Execution

CTE is a task-management framework that breaks down complex tasks into multiple subtasks and executes them step by step according to a logical sequence or dependency [21,22]. Unlike conventional chain of thought (CoT), which generates implicit, unstructured reasoning steps internally without external control, CTE explicitly decomposes RECIST assessment into four sequential, modular subtasks. Each step has predefined inputs, outputs, and logical dependencies, ensuring transparent and rule-compliant reasoning for longitudinal radiology report analysis. The CTE decomposes RECIST evaluation into four sequential subtasks, each designed with CoT prompts (Supplementary Materials Tables S1–S4, Figure S1), with logical dependencies:

Subtask 1: Lesion measurement extraction.

Input: Radiology reports of a single time point.

CoT-guided processing: The model first identifies target lesions (tumor long diameter ≥ 10 mm and lymph node short axis ≥ 15 mm) and non-target lesions (tumor long diameter < 10 mm and lymph node short axis < 15 mm), and it excludes normal structures (e.g., simple cysts < 10 mm and calcified lesions) per RECIST 1.1.

Output: Structured list of lesions (location, size, and type); subtask skipped if no measurable lesions (labeled “not evaluable [NE]”).

Subtask 2: SLD computation.

Input: Valid lesion list from Subtask 1.

Processing: Sum of long diameters of all target lesions, with RECIST 1.1 constraints: max 5 target lesions total, ≤2 per organ. For multi-modality reports (e.g., chest CT + abdominal MRI), lesions from different modalities are merged into a single SLD value.

Output: SLD value per time point; subtask skipped if <1 target lesion (labeled NE).

Subtask 3: New lesion detection.

Input: Lesion lists from consecutive time points (e.g., Time Point 1 vs. Time Point 2);

CoT-guided processing: Match lesions by location (e.g., “right upper lung mass” at Time Point 1 vs. Time Point 2); identify unreported lesions in the later time point and check if they meet target/non-target criteria.

Output: Binary “new lesion flag” (yes/no) and details of new lesions.

Subtask 4: Temporal comparison (response classification).

Input: SLD values from consecutive time points + new lesion flag;

Processing: Calculate percentage change in SLD vs. baseline and apply RECIST 1.1 criteria.

Output: Response category (CR/PR/SD/PD/NE) and rationale.

CR: Disappearance of target or non-target lesions;

PR: SLD reduction ≥ 30% (no new lesions);

SD: SLD reduction < 30% or increase < 20% (no new lesions);

PD: SLD increase ≥ 20% or new lesions;

NE: Insufficient information (e.g., <1 target lesion).

### 2.4. Measurement Extraction and Response Classification Validation

The performance of WiNGPT-32B and GPT-4 in lesion extraction was evaluated by GPT-4o, a State-of-the-Art multimodal LLM with demonstrated consistency in clinical text assessment [23], and verified by an expert panel, using a two-dimensional framework (Supplementary Materials Tables S1–S4): (1) consistency of lesion measurements (target lesion long diameter/lymph node short axis) with the original report; and (2) success rate of target lesion identification, where correct classification of lesions as “target/non-target/normal” per RECIST 1.1 was defined as successful.

The performance of 5-way classification (CR, PR, SD, PD, and NE) per RECIST 1.1 was assessed by three raters (WiNGPT-32B, GPT-4, and a radiologist with 8 years of experience). They independently evaluated de-identified radiology reports, where patient ID, date, and hospital name were removed to ensure blinding.

Three raters, including the compact LLM, GPT-4, and a radiologist with seven years of experience, independently evaluated radiological reports based on the RECIST criteria and determined the tumor response (CR/PR/SD/PD/NE). The reading results were then adjudicated by the above panel of three experienced radiologists as the reference standard. Figure 1B shows the evaluation framework.

For the above evaluation, the panel including three radiologists was considered. Among them, two radiologists with 8 and 13 years of experience independently validated the results, and discrepancies were resolved by a third radiologist with 25 years of experience.

### 2.5. Statistical Analysis

Continuous variables were tested for normality using the Kolmogorov–Smirnov test. Patient characteristics between the two hospitals were compared using the Mann–Whitney U test for non-normal continuous variables and χ^2^ test for categorical variables.

The extraction successful rates between WiNGPT-32B and GPT-4 were compared using the χ^2^ test. Taking the expert consensus as the reference standard, the following metrics were calculated for 5-category RECIST classification (CR/PR/SD/PD/NE): accuracy, recall, precision, and F1 score. The 95% confidence intervals (CIs) for accuracy were computed using Wilson’s method with continuity correction, while CIs for recall, precision, and F1 score were estimated via 1000 bootstrap resamples. Pairwise differences in accuracy between WiNGPT-32B, GPT-4, and the radiologist were tested using the McNemar test, with *p*-values adjusted via the Holm method to control type I error in multiple comparisons [24]. Inter-rater agreement was quantified using Cohen’s Kappa, with interpretation per Landis–Koch guidelines [25]: ≥0.81 as perfect agreement, 0.61–0.80 as substantial agreement, 0.41–0.60 as moderate, etc. Post hoc power was computed for pairwise accuracy comparisons using a two-proportion z-test for the overall cohort and for each RECIST category, and the McNemar test (asymptotic for small discordant counts) for paired subgroup comparisons (two-sided α = 0.05).

A two-tailed *p* < 0.05 was considered statistically significant. Analyses were performed using software (SPSS v25.0, IBM; RStudio v2024.09.1, Posit Software, Boston, MA, USA).

## 3. Results

### 3.1. Dataset Characteristics

**Figure 2:**
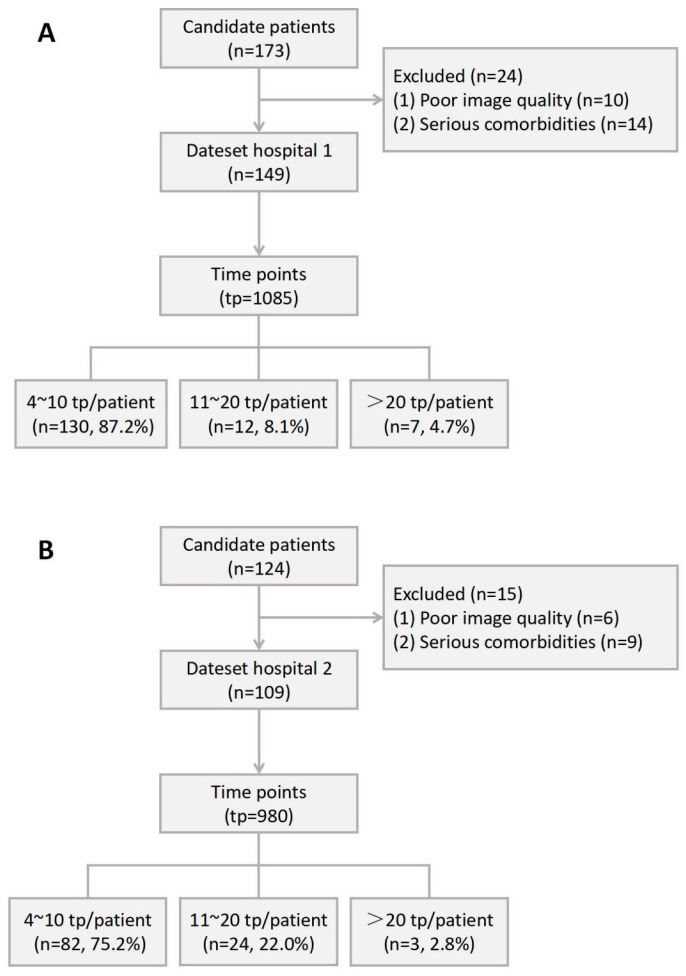
Patient-selection flowchart for solid-tumor patients across two hospitals. (**A**) Hospital 1: 173 candidate patients were screened, 24 were excluded, and 149 patients with 1085 imaging time points were included. (**B**) Hospital 2: 124 candidate patients were screened, 15 were excluded, and 109 patients with 980 imaging time points were included. Abbreviations: tp = time point.

A total of 258 patients (median age of 64.5 years [IQR 58–70 years], 176 males) with 2065 imaging time points were included (Table 1; Figure 2). Most patients (181/258, 70.2%) underwent both CT and MRI, 76 (29.5%) underwent CT alone, and only one (0.3%) underwent MRI alone. The number of imaging time points per patient aligned with RECIST 1.1’s recommended therapy monitoring interval (6 to 8 weeks) [5]: 212 patients (82.2%) completed 4–10 time points, 36 (13.9%) completed 11–20 time points, and 10 (3.9%) completed >20 time points.

Primary tumor types were dominated by lung cancer (106/258, 41.1%) and pancreatic cancer (66/258, 25.6%), followed by colorectal cancer (34/258, 13.2%) and liver cancer (22/258, 8.5%); other types (e.g., gastroesophageal and nasopharyngeal cancer) accounted for <10% of cases. Tumor-type distribution differed significantly between hospitals (*p* = 0.002): hospital 1 had a higher proportion of pancreatic cancer (34.9% vs. 12.8% in hospital 2), while hospital 2 had more lung-cancer cases (47.7% vs. 36.2% in hospital 1).

**Figure 3:**
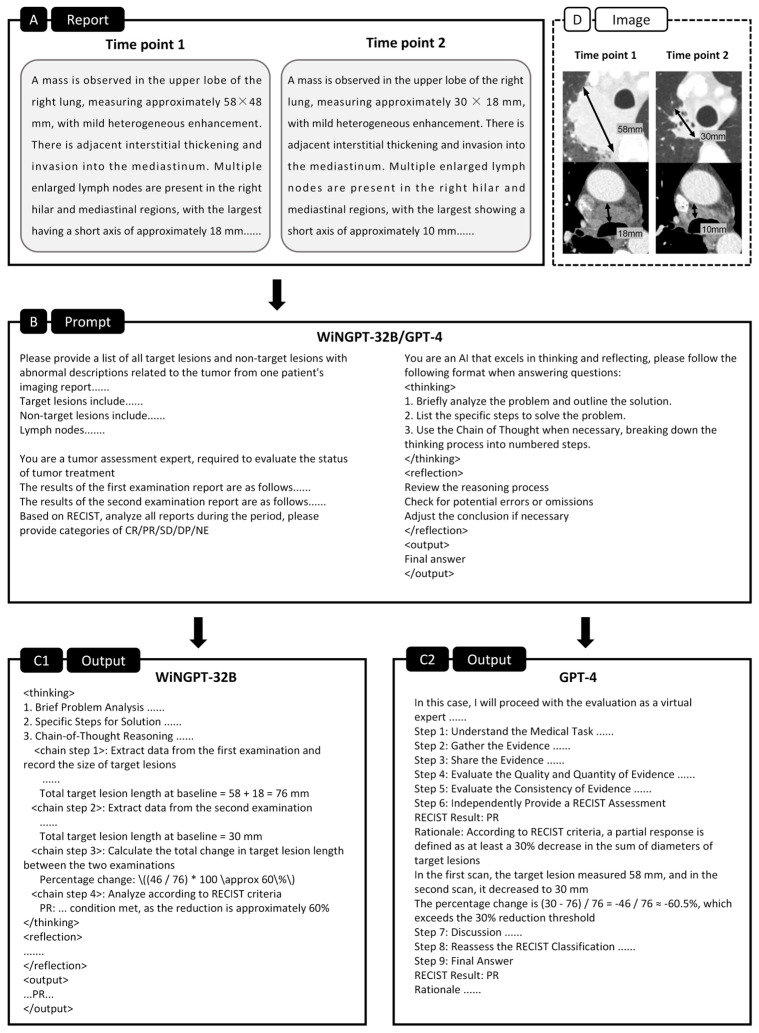
Exemplary workflow of the RECIST-based evaluation using WiNGPT and GPT-4 on longitudinal chest CT reports. (**A**) Input reports: Longitudinal chest CT reports from two time points were used as text input. The reports described changes in the primary lung lesion and lymph node measurements. (**B**) Prompt design: Structured prompts were provided to WiNGPT-32B and GPT-4 to guide target/non-target lesion extraction, SLD calculation, temporal comparison, and final RECIST classification. (**C1**,**C2**) Model outputs: WiNGPT-32B and GPT-4 generated stepwise responses, including extracted lesion measurements, calculated changes in tumor burden, and final response classification. In this example, both WiNGPT-32B and GPT-4 classified the tumor response as PR. (**D**) Image reference: CT images corresponding to the two reports; measurements represent the long diameter of the lung mass (upper) and the short axis of the lymph node (lower). CT images are shown for reference only; model assessment was based solely on radiology report text. See the Supplementary Tables S1–S4 and Figure S2 for additional details. Abbreviations: RECIST = Response Evaluation Criteria in Solid Tumors; PR = partial response.

A representative workflow of RECIST-based evaluation using LLMs is shown in Figure 3, with two detailed cases provided in Supplementary Materials Figures S2 and S3.

### 3.2. Lesion Measurement Extraction

The success rates of WiNGPT-32B and GPT-4 in extracting target lesions and lymph nodes are presented in Table 2, with accuracy validated by GPT-4o and confirmed by the expert panel. For target lesions, WiNGPT-32B achieved a success rate of 0.934 (95% CI: 0.922–0.944), slightly higher than GPT-4’s 0.920 (0.907–0.931; *p* = 0.083). For lymph nodes meeting target criteria, both models exhibited excellent performance: WiNGPT-32B achieved a success rate of 0.986 (0.980–0.990), and GPT-4 reached 0.988 (0.982–0.992; *p* = 0.584).

### 3.3. RECIST Classification Evaluation

The performance of WiNGPT-32B, GPT-4, and the 8-year-experience radiologist in five-category RECIST classification (CR/PR/SD/PD/NE) is summarized in Table 3 and Table 4, with reference to the expert panel’s consensus.

WiNGPT-32B achieved an overall accuracy of 0.805 (95% CI: 0.786–0.823), which was significantly higher than GPT-4’s 0.699 (0.678–0.720; *p* < 0.001), but lower than the radiologist’s 0.915 (0.901–0.928; *p* < 0.001). Consistent trends were observed for secondary metrics: WiNGPT-32B (recall = 0.805, precision = 0.804, F1 = 0.803) outperformed GPT-4 (recall = 0.702, precision = 0.717, F1 = 0.693) but was inferior to the radiologist (recall = 0.915, precision = 0.917, F1 = 0.915).

The inter-rater agreement analysis showed moderate agreement between WiNGPT-32B and GPT-4 (Cohen’s Kappa: 0.409; 95% CI: 0.379–0.439) and substantial agreement between WiNGPT-32B and the radiologist (0.630, 0.602–0.659) (Table S2). The confusion matrix (Table S3) revealed the distribution of RECIST classification outcomes across all five categories.

The performance across all five categories of the RECIST response is shown in Table 4 and Table S4. For CR, WiNGPT-32B achieved an accuracy of 0.600 (95%CI: 0.274–0.863), which was lower than the radiologist’s 0.900 (0.541–0.995) (*p* = 0.375) but higher than GPT-4’s 0.100 (0.005–0.459) (*p* = 0.125). For PR, WiNGPT-32B achieved a high accuracy of 0.850 (0.809–0.884), with no significant difference from the radiologist’s 0.886 (0.847–0.915) (*p* = 0.223), and outperformed GPT-4 in terms of F1 score (0.808, 0.770–0.847 vs. 0.731, 0.684–0.778). In the SD category, WiNGPT-32B showed an accuracy of 0.789 (0.750–0.822), lower than the radiologist’s 0.947 (0.923–0.964) (*p* < 0.001), but higher than GPT-4’s 0.675 (0.632–0.715) (*p* < 0.001). Notably, for PD (clinical critical endpoint), WiNGPT-32B exhibited strong performance, achieving an accuracy of 0.804 (0.772–0.833), recall of 0.804 (0.775–0.834), precision of 0.882 (0.856–0.907), and F1 score of 0.841 (0.813–0.870). This F1 score was significantly higher than GPT-4’s 0.755 (0.720–0.790) (*p* < 0.001) and approached the radiologist’s 0.922 (0.902–0.942) (*p* = 0.002). Finally, for the NE category, WiNGPT-32B’s accuracy was 0.778 (0.718–0.828), lower than the radiologist’s 0.880 (0.830–0.918) (*p* = 0.004), but comparable to GPT-4’s 0.803 (0.745–0.851) (*p* = 0.602).

The primary analyses were well powered. Based on 2065 time-point evaluations, the study had >99.9% power to detect the observed difference in overall RECIST classification accuracy between WiNGPT-32B and the radiologist. Category-specific power varied according to the number of reference-positive evaluations. CR had only 6% power to detect a 10-percentage-point accuracy difference, and NE had borderline power of 69%. In contrast, PR, SD, and PD were adequately powered, with power estimates of 93%, 96%, and 99%, respectively.

## 4. Discussion

This study successfully constructed a 32-billion-parameter LLM for RECIST-based tumor response evaluation using longitudinal radiology reports, achieving an overall accuracy of 0.805, surpassing GPT-4 (0.699) and narrowing the gap with human radiologists (0.915).

Recent work supports the feasibility of AI-assisted RECIST classification from radiology reports. Mottin et al. compared traditional machine-learning and fine-tuned pretrained language models using German radiology reports, achieving F1 scores of 0.90 (binary classification) and 0.86 (four-category), demonstrating the cross-lingual and cross-institutional applicability of domain-adapted pretrained models [26]. Park et al. further evaluated Gemma models for RECIST assessment on real-world oncology reports, attaining an F1 score of 0.815 in for RECIST classification and showing that LLMs can perform guideline-aligned clinical reasoning from narrative documents [27]. A recent systematic review of AI-driven RECIST-based treatment response assessment noted that report-derived models generally yield higher classification accuracy, while image-segmentation models excel in lesion delineation [28]. These findings support the validity of our report-based approach. While Tordjman et al. reported RECIST classification accuracies of 0.74 (DeepSeek-R1) and 0.72 (Llama 3.1-405B), their evaluation was limited to four categories and two consecutive reports [14]. In comparison, our study addresses five-category assessment using longitudinal reports, and WiNGPT-32B is designed for local deployment. Our model achieves 0.805 overall accuracy, offering a practical, privacy-preserving solution for clinical workflows. The favorable performance of our model may stem from two core innovations: first, the CTE framework decomposed complex RECIST tasks into modular subtasks; second, the 100 expert-validated distillation samples served as a high-quality RECIST-specific seed set and likely helped transfer structured reasoning patterns to WiNGPT-32B, thereby improving its downstream RECIST performance. The synergy between the CTE framework and GPT-4-based knowledge distillation further explains WiNGPT-32B’s superior performance. The modular decomposition of CTE provides a clear “reasoning roadmap” for the distilled RECIST knowledge, ensuring that the model applies clinical rules precisely at each subtask stage, rather than treating RECIST assessment as a single unstructured text task. This is particularly evident in PD classification, where WiNGPT-32B’s F1 score (0.841) significantly outperforms GPT-4 (0.755).

To delve deeper into the source of LLM’s advantage, we employed GPT-4o as an independent evaluation benchmark because of its strong consistency in subjective rating tasks and multimodal reasoning capabilities, which make it ideal for validating clinical AI performance [29]. Shahriar et al. further confirmed GPT-4o’s proficiency in complex text evaluation, supporting its use as a benchmark for clinical artificial intelligence text and language evaluation [30]. Both WiNGPT-32B and GPT-4 achieved high success rates in extracting target and lymph node lesions (target lesions: 0.934 vs. 0.920; lymph nodes: 0.986 vs. 0.988), with no statistically significant difference (*p* > 0.001). This indicates that the overall improvement in tumor response assessment does not originate from lesion identification alone, but rather from the downstream multi-step reasoning capabilities optimized by the CTE framework. The lower performance of WiNGPT-32B in CR classification may be related to the small number of CR cases and the fact that complete disappearance of all target and non-target lesions is not always explicitly documented in routine radiology reports.

This downstream reasoning advantage directly translates to substantial clinical utility, addressing the logical reasoning limitations of conventional LLMs highlighted by Hagendorff et al. [31]. The CTE framework’s modular decomposition enables structured multi-step reasoning that aligns with routine radiology workflows. For PD, a critical trigger for treatment modification, WiNGPT-32B achieved a 19.6% missed diagnosis rate (551/694 correct cases), outperforming GPT-4 (30.7%). This reduction in missed PD classifications may have greater clinical impact than equivalent numerical improvements in less urgent categories, such as SD, because PD often directly prompts treatment modification. It correctly identified new lesions via the CTE framework, enabling timely PD classification; this is invaluable for busy clinics, where radiologists face heavy longitudinal assessment burdens.

Additionally, the 32B-parameter open-source design supports local deployment on hospital GPUs (2 × Titan RTX), addressing patient data privacy concerns (a key limitation of closed-source LLMs like GPT-4) and reducing assessment time, facilitating integration into Picture Archiving and Communication Systems (PACS). In this setting, WiNGPT-32B can assist clinicians by automatically analyzing radiology reports and providing preliminary RECIST classifications, which not only reduce cognitive burden but also help reinforce confidence in their own clinical judgments. Similarly, McDuff et al. showed that Articulate Medical Intelligence Explorer, an LLM optimized for diagnostic reasoning, can significantly improve clinicians’ diagnostic performance and decision quality [32]. Zhu et al. demonstrated that an oncology-specific LLM could accurately predict cancer progression across multiple cancer types and institutions, underscoring its potential for integration into routine oncology workflows [33].

Our study has several limitations. First, the model relies on radiology reports rather than raw imaging data. While raw image inputs would be ideal, text-only LLMs are currently more deployable in clinical workflows because radiology reports are more readily accessible in longitudinal follow-up practice. Second, sample representativeness is limited. Data from two cohorts: Lung and pancreatic cancers accounted for 66.7% of cases, while rare tumor types (e.g., nasopharyngeal cancer, <2.3%) were underrepresented, which may limit the broader applicability of the findings. Although the primary overall analyses were adequately powered, statistical power was limited for some less frequent RECIST categories. In particular, the small number of CR evaluations resulted in less stable category-specific estimates. The low frequency of CR in our cohort is consistent with the class imbalance observed in previous real-world RECIST studies. For example, Feinberg et al. reported that CR accounted for only 4.8% of 956 real-world RECIST assessments across multiple solid tumor types [34]. In our cohort, CR was even rarer, leading to limited statistical power and less stable estimates. Future studies should expand clinical validation through prospective multicenter cohorts that include a wider range of tumor types and more diverse imaging settings to further improve generalizability. Third, the evaluation focused exclusively on time point-based RECIST classification, without incorporating longitudinal endpoints such as best overall response, which require synthesizing information across multiple time points and integrating clinical context, such as treatment regimens and physician judgment. Finally, variability in radiology report styles and linguistic expressions may affect generalizability and stability. Despite being trained on a wide range of medical knowledge, the model may still face non-standardized or ambiguous descriptions, potentially impacting classification consistency.

## 5. Conclusions

This study developed WiNGPT-32B integrated with a CTE framework for RECIST-based tumor response evaluation using longitudinal radiology reports. It showed higher overall RECIST classification accuracy than GPT-4 and favorable performance in PD detection, supported by CTE’s modular RECIST task decomposition, but it remained inferior to radiologists. Open-source designs that allow local deployment can alleviate patient data privacy concerns and improve workflow efficiency. The results indicate that the combination of large language models and the CTE framework offers a feasible option to support automated RECIST evaluation.

## Figures and Tables

**Table 1:** Demographic and clinical features of included solid-tumor patients.

Characteristics		Overall (*n* = 258)	Hospital 1 (*n* = 149)	Hospital 2 (*n* = 109)	*p*-Value
Age, years [IQRs]		64.5 [58, 70]	64 [58, 70]	65 [58, 71]	0.743
Sex					0.004
	Male, *n* (%)	176 (68.2%)	91 (61.1%)	85 (78.0%)	
	Female, *n* (%)	82 (31.8%)	58 (38.9%)	24 (22.0%)	
Examination type					0.653
	CT + MRI, *n* (%)	181 (70.2%)	103 (69.1%)	78 (71.6%)	
	CT, *n* (%)	76 (29.5%)	45 (30.2%)	31 (28.4%)	
	MRI, *n* (%)	1 (0.3%)	1 (0.7%)	0	
	Time points				0.005
	4~10, *n* (%)	212 (82.2%)	130 (87.2%)	82 (75.2%)	
	11~20, *n* (%)	36 (13.9%)	12 (8.1%)	24 (22.0%)	
	>20, *n* (%)	10 (3.9%)	7 (4.7%)	3 (2.8%)	
Primary tumor site					0.002
	Lung, *n* (%)	106 (41.1%)	54 (36.2%)	52 (47.7%)	
	Pancreas, *n* (%)	66 (25.6%)	52 (34.9%)	12 (12.8%)	
	Colorectum, *n* (%)	34 (13.2%)	20 (13.4%)	14 (12.8%)	
	Liver, *n* (%)	22 (8.5%)	8 (5.4%)	14 (12.8%)	
	Gastroesophagus, *n* (%)	14 (5.4%)	6 (4.0%) 8	8 (7.3%)	
	Nasopharynx, *n* (%)	6 (2.3%)	3 (2.0%)	3 (2.8%)	
	Larynx, *n* (%)	2 (0.8%)	1 (0.7%)	1 (0.9%)	
	Bone, *n* (%)	3 (1.2%)	3 (2.0%)	0	
	Bladder, *n* (%)	2 (0.8%)	2 (1.3%)	0	
	Mediastinum, *n* (%)	2 (0.8%)	0	2 (1.8%)	
	Renal, *n* (%)	1 (0.4%)	0	1 (0.9%)	

Note. Categorical variables are reported as the number of patients with percentages in parentheses, continuous variables are reported as medians with interquartile ranges (IQRs) in parentheses, and *p*-values for comparisons of patient characteristics between the two hospitals were performed using the Mann–Whitney U test for non-normal continuous variables and the chi-square (χ^2^) test for categorical variables.

**Table 2:** Comparison of target lesions and lymph node lesions extraction success rates between WiNGPT-32B and GPT-4.

	WiNGPT-32B	GPT-4	*p*-Value
Target lesions	0.934 (0.922–0.944)	0.920 (0.907–0.931)	0.083
Lymph node lesions	0.986 (0.980–0.990)	0.988 (0.982–0.992)	0.584

Note. This table presents the success rates of WiNGPT-32B and GPT-4 in extracting two types of RECIST 1.1-compliant lesions: (1) target lesions (tumor long diameter ≥ 10 mm) and (2) lymph nodes meeting target criteria (short axis ≥ 15 mm). All extraction success rates were validated for accuracy by GPT-4o and further confirmed by an expert panel. The 95% confidence intervals (CIs) for success rates were calculated using Wilson’s method with continuity correction, and *p*-values for comparisons of success rates between WiNGPT-32B and GPT-4 were performed using the chi-square (χ^2^) test.

**Table 3:** Overall performance of WiNGPT-32B, GPT-4, and a radiologist in 5-category RECIST classification.

	Accuracy	Recall	Precision	F1 Score
WiNGPT-32B	0.805 (0.786–0.823)	0.805 (0.801–0.807)	0.804 (0.802–0.805)	0.803 (0.801–0.805)
GPT-4	0.699 (0.678–0.720)	0.702 (0.695–0.709)	0.717 (0.715–0.720)	0.693 (0.689–0.696)
Radiologist	0.915 (0.901–0.928)	0.915 (0.914–0.917)	0.917 (0.916–0.918)	0.915 (0.914–0.915)

Note. The overall performance of 5 categories included complete response (CR), partial response (PR), stable disease (SD), progressive disease (PD), and not evaluable (NE). All metrics are calculated using the consensus of three radiologists as the reference standard. The 95% confidence intervals (CIs) for accuracy were computed via Wilson’s method with continuity correction. For recall, precision, and F1 score, 95% CIs were estimated through 1000 bootstrap resamples. Abbreviations: RECIST = Response Evaluation Criteria in Solid Tumors.

**Table 4:** Per-category RECIST classification performance metrics among WiNGPT-32B, GPT-4, and an 8-year-experience radiologist.

	Accuracy	Recall	Precision	F1 Score
WiNGPT-32B				
CR	0.600 (0.274–0.863)	0.600 (0.296–0.904)	0.333 (0.116–0.551)	0.429 (0.200–0.657)
PR	0.850 (0.809–0.884)	0.850 (0.814–0.887)	0.770 (0.729–0.811)	0.808 (0.770–0.847)
SD	0.789 (0.750–0.822)	0.789 (0.753–0.824)	0.798 (0.763–0.833)	0.793 (0.758–0.829)
PD	0.804 (0.772–0.833)	0.804 (0.775–0.834)	0.882 (0.856–0.907)	0.841 (0.813–0.870)
NE	0.778 (0.718–0.828)	0.778 (0.725–0.831)	0.717 (0.661–0.772)	0.746 (0.692–0.799)
GPT-4				
CR	0.100 (0.005–0.459)	0.100 (0.000–0.286)	0.500 (0.000–1.000)	0.167 (0.000–0.683)
PR	0.706 (0.656–0.751)	0.706 (0.659–0.752)	0.757 (0.712–0.803)	0.731 (0.684–0.778)
SD	0.675 (0.632–0.715)	0.675 (0.635–0.716)	0.765 (0.726–0.804)	0.717 (0.676–0.759)
PD	0.693 (0.657–0.728)	0.693 (0.659–0.728)	0.829 (0.798–0.860)	0.755 (0.720–0.790)
NE	0.803 (0.745–0.851)	0.803 (0.752–0.854)	0.428 (0.382–0.475)	0.559 (0.512–0.605)
Radiologist				
CR	0.900 (0.541–0.995)	0.900 (0.714–1.000)	0.900 (0.715–1.000)	0.900 (0.714–1.000)
PR	0.886 (0.847–0.915)	0.886 (0.853–0.918)	0.959 (0.938–0.980)	0.921 (0.892–0.949)
SD	0.947 (0.923–0.964)	0.947 (0.928–0.967)	0.861 (0.833–0.890)	0.902 (0.878–0.927)
PD	0.920 (0.896–0.938)	0.920 (0.899–0.940)	0.924 (0.904–0.944)	0.922 (0.902–0.942)
NE	0.880 (0.830–0.918)	0.880 (0.839–0.922)	0.963 (0.937–0.988)	0.920 (0.883–0.956)

Note. This table shows the category-specific performance for each RECIST response category. The 95% confidence intervals (CIs) for accuracy were computed via Wilson’s method with continuity correction. For recall, precision, and F1 score, 95% CIs were estimated through 1000 bootstrap resamples. Abbreviations: RECIST = Response Evaluation Criteria in Solid Tumors; CR = complete response; PR = partial response; SD = stable disease; PD = progressive disease; NE = not evaluable.

## Data Availability

The raw data supporting the conclusions of this article will be made available by the corresponding author upon request.

## Supplementary Materials

The following supporting information can be downloaded at https://www.mdpi.com/article/10.3390/diagnostics16132020/s1, Table S1: Diverse datasets used for model training. Table S2: Inter-observer agreement for 5-category classification based on the RECIST criteria. Table S3: Confusion matrix of WINGPT, GPT-4, and radiologist’s classification results based on RECIST criteria. Table S4: Classification performance for each category of the RECIST in hospitals 1 and 2. Figure S1: Classification workflow based on RECIST criteria. Figure S2: A patient with lung cancer who was treated with chemotherapy. Figure S3: A patient with colon cancer who was treated with chemotherapy and targeted therapy.

## Abbreviations

The following abbreviations are used in this manuscript:

RECIST	Response Evaluation Criteria in Solid Tumors
LLM	large language model
SLD	sum of length diameters
CTE	chained task execution
CoT	chain of thought
RoPE	Rotary Positional Embedding
RMSNorm	Root Mean Square Layer Normalization
SwiGLU	Swish–Gated Linear Unit
CR	complete response
PR	partial response
SD	stable disease
PR	progressive disease
NE	not evaluable
PACS	Picture Archiving and Communication Systems
