# Supplementary material for: WiNGPT-32B: An Open-Source, Locally Deployable LLM for RECIST Assessment via Chained Task Execution Using Radiology Report Text
Source: Diagnostics (Basel). 2026 Jun 28;16(13):2020. doi: 10.3390/diagnostics16132020 (PMC13360225; doi:10.3390/diagnostics16132020)
Supplement: Supplementary file 1 [file diagnostics-16-02020-s001.zip › diagnostics-4226011-supplementary.pdf]

Supplementary Materials

Methods

Table S1: Diverse datasets used for model training

Table S2: Inter-observer agreement for 5-category classification based on the RECIST criteria

Table S3: Confusion matrix of WINGPT, GPT-4, and radiologist's classification results based on RECIST criteria

Table S4: Classification performance for each category of the RECIST in hospitals 1 and 2

Figure S1: Classification workflow based on RECIST criteria

Figure S2: A patient with lung cancer who was treated with chemotherapy

Figure S3: A patient with colon cancer who was treated with chemotherapy and targeted therapy

Methods

Model training

The data used for training consisted of the following components: auxiliary diagnostic data (6,000 instances), quality control result extraction data (2,000), examination report generation data (180,000), medical question-and-answer data (56,000), and information extraction data (3,500) (Table S1). This diverse dataset ensures robust model training across diagnostic accuracy, operational compliance, and clinical documentation tasks.

The pre-training process leveraged the open-source general-purpose model Qwen2.5-32B (arXiv:2412.15115). The pre-training dataset size of this model was expanded from 7T tokens to 18T tokens. With the introduction of Qwen2.5-math technology, the mathematical reasoning performance was also improved. Qwen2.5 has been featured with a transformer architecture enhanced by Rotary Positional Embeddings (RoPE) (Neurocomputing 2024,568:127063), alongside optimized components like RMSNorm (arXiv:1910.07467v1) and SwiGLU (arXiv:2002.05202) functions.

Rotary Positional Embeddings (RoPE) incorporates relative positional information by rotating query and key matrices with angle-based transformations, effectively capturing long-range dependencies while maintaining computational efficiency.

Root Mean Square Layer Normalization (RMSNorm) represents an enhanced layer normalization method primarily engineered to stabilize neural network training process. Unlike conventional LayerNorm's reliance on mean and variance calculations, RMSNorm achieves normalization through sophisticated root mean square computations of input features. Compared to traditional methods, RMSNorm offers more efficient computational performance and exhibits superior gradient stability in large-scale language models.

SwiGLU is a hybrid activation function that integrates the nonlinear smoothness of Swish activation function with the structural advantages of Gated Linear Unit (GLU). This architectural marvel employs a dynamic gating mechanism to regulate information flow while leveraging Swish's curvilinear properties to amplify model representational capacity. Particularly adept at harmonizing expressive power with operational efficiency, SwiGLU has become an indispensable component in both pre-training paradigms and fine-tuning workflows for language models.

$$SwiGLU(x, W, V, b, c) = Swish_1(xW + b) \otimes (xV + c)$$

The model was fine-tuned on our specialized dataset, ensuring mastery in interpreting and analyzing diverse medical data. During the fine-tuning process, we deployed techniques such as data augmentation and dropout regularization to decrease overfitting. By integrating chains of thought into its cognitive architecture, the model was guided to generate more coherent and logical predictions, thereby enhancing its performance in complex clinical scenarios.

Data Distillation Process Using Teacher-Student Framework

In this study, we employed a knowledge distillation (arXiv:1503.02531v1) approach for data generation, using GPT-4 as the teacher model and WiNGPT as the student model. The teacher model was utilized to generate predictions on the dataset, producing outputs that encapsulate rich information from the teacher's learned representations. Subsequently, the student model was trained to replicate the labels provided by the teacher model. The training process for the student model was guided by cross-entropy loss, enabling the student to effectively mimic the teacher's outputs.

Optimizing RECIST Information Extraction via Data Distillation

To enhance the extraction of RECIST-related information, an independent subset of 100 radiological reports was selected for few-shot learning. The teacher model was employed to generate soft labels for this data, which underwent rigorous manual review and verification. Following systematic refinement of the distilled data, the annotated outputs from the teacher model were leveraged to train the student model WiNGPT, thereby ensuring enhanced performance in information extraction tasks. The latest WiNGPT is available at wingpt.winning.com.cn

Chained task execution

Chained Task Execution (CTE) is a pattern that connects multiple tasks in a specific order for execution, where the execution of each task depends on the completion or output of the preceding task. This pattern is widely applied in fields

such as programming, automated workflows, and distributed systems, aiming to achieve logically clear and highly collaborative task management. In this study, CTE provides a structured approach to task management through well-defined RECIST-based tumor assessment task dependencies and data flows. The key lies in the rational design of task sequences, handling dependencies and errors. This research employs multi-step reasoning through modular task decomposition:

Lesion measurement extraction: Input = radiology reports; Output = structured list of target lesions (long diameter > 10 mm), non-target lesions (e.g., lymph nodes with short axis 10 - 14.9 mm), and lymph nodes (short axis > 15 mm); subtask skipped if no measurable lesions are described (final response labeled as “not evaluable [NE]”).

SLD computation: Input = valid lesion list from Subtask 1; Output = SLD value (sum of long diameters of all target lesions) per time point; subtask skipped if < 1 target lesion is identified (final response labeled as NE).

New lesion detection: Input = lesion lists from consecutive time points; Output = binary “new lesion flag” (yes/no) based on whether previously unreported lesions (meeting target/non-target criteria) are identified.

Temporal comparison: Input = SLD values from consecutive time points + new lesion flag; Output = time-point response category (complete response [CR]/partial response [PR]/stable disease [SD]/progressive disease [PD]/NE) per RECIST 1.1.

RECIST report generation: Input = response category from Subtask 4 + lesion details from Subtask 1; Output = structured RECIST report (including lesion measurements, SLD changes, and response rationale).

Chain of Thought (CoT) is a prompt engineering technique designed to guide large language models in step-by-step reasoning to solve complex problems. The emphasis of CoT lies in enhancing the model's logical reasoning ability and interpretability through explicit intermediate reasoning steps.

From a relational perspective, both approaches involve decomposing complex problems. CoT can then be embedded within CTE. Strictly speaking, our framework integrates CoT into CTE.

Hardware

The compact LLM was trained on a Linux platform (Ubuntu 20.04, Canonical Ltd.) with 8 graphics processing units (GPUs) (A800, Nvidia) with 640 GB of graphics random-access memory, and was inferenced on 2 GPUs (Titan RTX, Nvidia) with 48 GB of memory.

Engineering prompts for WiNGPT or GPT-4 to generate structured outputs as follows:

Please provide a list of all target lesions and non-target lesions with abnormal descriptions related to the tumor from one patient's imaging report, giving the larger of the two size measurements for the target lesions as the major axis length. Lymph nodes should be listed separately, with the lesion measurements being the shorter of the two size measurements as the minor axis length. If there is no description, please state "not described."

Target lesions include:

Lesions with a long diameter greater than 10 mm

Malignant lymph nodes with a short diameter greater than 15 mm

A maximum of 2 target lesions per organ, with a total not exceeding 5\n\n

Non-target lesions include:

Lymph nodes with a short axis of 10-14.9 mm

Pleural effusion, pericardial effusion, ascites, inflammatory breast diseases, clinical lesions that cannot be accurately identified.

Bone abnormalities, calcified lesions are not non-target lesions

Note: Nodules less than 10mm are considered normal, cysts are not target lesions or non-target lesions.
Lesions without abnormalities are not described.

Format Example:
Target Lesion| Description| Long Diameter (Larger)
Non-target Lesion| Description

Tumor Progression Comparison
You are a tumor assessment expert, required to evaluate the status of tumor treatment. The following are tumor examination reports:
The results of the first examination report are as follows: Information extraction results from the first report.
The results of the second examination report are as follows: Information extraction results from the second report;
Based on RECIST, analyze all reports during the period, please provide categories of complete remission, partial remission, stable disease, or disease progression, among which:
Calculate the change in the sum of the lengths of the target lesions from the two examinations.
- Complete remission, all target lesions have completely disappeared.
- Partial remission, the total length of the target lesions has decreased by 30% or more.
- Stable disease, the total length of the target lesions has decreased by less than 30% or increased by less than 20%, and no new lesions have appeared.
- If there are unexamined situations, mark as not evaluable.
- Disease progression, the total length of the lesions has increased by 20% or more, or new lesions have appeared.

You are an AI that excels in thinking and reflecting, please follow the following format when answering questions:
<thinking>
1. Briefly analyze the problem and outline the solution.
2. List the specific steps to solve the problem.
3. Use the Chain of Thought when necessary, breaking down the thinking process into numbered steps.
</thinking>
<reflection>
Review the reasoning process
Check for potential errors or omissions
Adjust the conclusion if necessary
</reflection>
<output>
Final answer
</output>

Instructions:
- All labels must be on separate lines, containing no other text.
- Provide detailed explanations for each reasoning step, breaking down complex problems into combinations of simple problems.
- Use an analytical, somewhat formal tone to clearly convey the thought process.

Engineering prompts of GPT-4o for assessing the accuracy of WiNGPT and GPT-4 in extracting target lesions and lymph node lesions as follows:

Information extraction results: [text of information extraction results]

Original report: [Text of the original report results]

The above are the information extraction results for the patient's target lesions and lymph nodes. Please analyze the following results:

1. Is the measurement of the target lesion and lymph accurate;
2. Is the identification of the target lesion accurate, returned in json format, example return form

{ "target lesion": { "accurate": true, "reason": "The location and size of the target lesion are consistent with the original report results" }, "lymph": {"accurate":true,"reason": The lymph description is consistent with the original report results}}

Table S1: Diverse datasets used for model training

Data	Instance, *n*	Content
Auxiliary diagnostic data	6000	Using a patient's basic information, chief complaint, present illness history, past medical history, and laboratory or imaging tests to infer a patient's diagnosis.
Quality control result extraction data	2000	Using specified electronic medical record data, determine whether the quality control results are accurate according to specific electronic medical record quality control rules.
Examination report generation data	180000	Generating examination impression based on findings from medical imaging studies, including CT, MR, ultrasound and endoscopy examination.
Medical question-and-answer data	56000	medical question-answering dataset
Information extraction data	3500	Required medical indicators are extracted from medical texts according to the specified format.

Table S2: Inter-observer agreement for 5-category classification based on the RECIST criteria

	Overall (n=258)	Hospital 1 (n=149)	Hospital 2 (n=109)
WiNGPT vs. GPT-4, Kappa (95%CI)	0.409 (0.379-0.439)	0.390 (0.348-0.432)	0.422 (0.379-0.464)
WiNGPT vs. Radiologist, Kappa (95%CI)	0.630 (0.602-0.659)	0.665 (0.628-0.701)	0.580 (0.537-0.624)

The 95% confidence intervals were estimated based on the standard error using the normal approximation method.

Table S3: Confusion matrix of WINGPT, GPT-4, and radiologist's classification results based on RECIST criteria

		Overall					Hospital 1					Hospital 2				
		CR	PR	SD	PD	NE	CR	PR	SD	PD	NE	CR	PR	SD	PD	NE
WiNGPT	CR	**6**	5	1	0	6	**1**	3	0	0	2	**5**	2	1	0	4
	PR	1	**312**	39	43	10	0	**187**	15	18	6	1	**125**	24	25	4
	SD	0	19	**403**	57	26	0	16	**213**	37	11	0	3	**190**	20	15
	PD	2	9	53	**551**	10	0	2	21	**198**	4	2	7	32	**353**	6
	NE	1	22	15	34	**182**	1	19	14	34	**134**	0	3	1	0	**48**
GPT-4	CR	**1**	1	0	0	0	**1**	1	0	0	0	**0**	0	0	0	0
	PR	0	**259**	36	30	17	0	**159**	25	12	16	0	**100**	11	18	1
	SD	4	25	**345**	63	14	0	14	**186**	15	8	4	11	**159**	48	6
	PD	0	18	65	**475**	15	0	15	28	**214**	9	0	3	37	**261**	6
	NE	5	64	65	117	**188**	1	38	24	46	**124**	4	26	41	71	**64**
Radiologist	CR	**9**	0	0	0	1	**2**	0	0	0	1	**7**	0	0	0	0
	PR	0	**325**	4	4	6	0	**214**	2	3	4	0	**111**	2	1	2
	SD	1	23	**484**	47	7	0	6	**251**	1	4	1	17	**233**	46	3
	PD	0	19	19	**630**	14	0	7	10	**282**	12	0	12	9	**348**	2
	NE	0	0	4	4	**206**	0	0	0	1	**136**	0	0	4	3	**70**
		Reference standard														

CR, complete response; PR, partial response; SD, stable disease; PD, progressive disease; NE, not evaluable

Table S4: Classification performance for each category of the RECIST in hospitals 1 and 2

	Hospital 1				Hospital 2			
	Accuracy	Recall	Precision	F1 score	Accuracy	Recall	Precision	F1 score
WiNGPT								
CR	0.500 (0.027-0.973)	0.500 (0.100-0.900)	0.167 (0.000-0.465)	0.250 (0.000-0.596)	0.625 (0.259-0.898)	0.625 (0.351-0.899)	0.417 (0.138-0.696)	0.500 (0.217-0.783)
PR	0.824 (0.767-0.870)	0.824 (0.774-0.873)	0.827 (0.778-0.877)	0.826 (0.776-0.875)	0.893 (0.827-0.937)	0.893 (0.848-0.938)	0.698 (0.631-0.766)	0.784 (0.723-0.844)
SD	0.810 (0.756-0.854)	0.810 (0.764-0.856)	0.769 (0.719-0.819)	0.789 (0.741-0.837)	0.766 (0.707-0.816)	0.766 (0.711-0.821)	0.833 (0.785-0.882)	0.798 (0.746-0.850)
PD	0.690 (0.632-0.742)	0.690 (0.629-0.750)	0.880 (0.838-0.922)	0.773 (0.719-0.828)	0.887 (0.851-0.916)	0.887 (0.856-0.918)	0.883 (0.851-0.914)	0.885 (0.853-0.916)
NE	0.854 (0.786-0.903)	0.854 (0.805-0.902)	0.663 (0.598-0.729)	0.747 (0.687-0.807)	0.623 (0.505-0.729)	0.623 (0.492-0.755)	0.923 (0.851-0.996)	0.744 (0.626-0.863)
GPT-4								
CR	0.500 (0.027-0.973)	0.500 (0.000-1.000)	0.500 (0.000-1.000)	0.500 (0.000-1.000)	NA	NA	NA	NA
PR	0.700 (0.636-0.758)	0.700 (0.639-0.762)	0.750 (0.692-0.808)	0.714 (0.664-0.785)	0.714 (0.631-0.786)	0.714 (0.637-0.792)	0.769 (0.697-0.842)	0.741 (0.665-0.816)
SD	0.707 (0.648-0.761)	0.707 (0.648-0.767)	0.834 (0.785-0.883)	0.641 (0.578-0.700)	0.697 (0.633-0.755)	0.641 (0.579-0.703)	0.697 (0.638-0.757)	0.668 (0.607-0.729)
PD	0.746 (0.690-0.794)	0.746 (0.693-0.798)	0.805 (0.757-0.852)	0.774 (0.724-0.824)	0.656 (0607-0.702)	0.656 (0.603-0.709)	0.850 (0.810-0.890)	0.740 (0.691-0.789)
NE	0.790 (0.716-0.849)	0.790 (0.737-0.842)	0.532 (0.468-0.596)	0.636 (0.574-0.698)	0.831 (0.725-0.904)	0.831 (0.780-0.882)	0.311 (0.247-0.374)	0.452 (0.384-0.520)
Radiologist								
CR	1.000 (0.198-1.000)	1.000 (1.000-1.000)	0.667 (0.133-1.000)	0.800 (0.347-1.000)	0.875 (0.467-0.993)	0.875 (0.630-1.120)	1.000 (1.000-1.000)	0.933 (0.749-1.000)
PR	0.943 (0.902-0.968)	0.943 (0.912-0.973)	0.960 (0.934-0.985)	0.951 (0.923-0.979)	0.793 (0.714-0.855)	0.793 (0.719-0.867)	0.793 (0.719-0.867)	0.793 (0.719-0.867)
SD	0.954 (0.920-0.975)	0.954 (0.929-0.980)	0.958 (0.934-0.982)	0.956 (0.931-0.981)	0.940 (0.900-0.965)	0.940 (0.913-0.966)	0.940 (0.913-0.966)	0.940 (0.913-0.966)
PD	0.983 (0.957-0.994)	0.983 (0.968-0.997)	0.907 (0.874-0.939)	0.943 (0.917-0.969)	0.874 (0.837-0.905)	0.874 (0.841-0.908)	0.874 (0.841-0.908)	0.874 (0.841-0.908)
NE	0.866 (0.801-0.913)	0.866 (0.809-0.923)	0.993 (0.978-1.000)	0.925 (0.881-0.969)	0.909 (0.816-0.960)	0.909 (0.845-0.973)	0.909 (0.845-0.973)	0.909 (0.845-0.973)

Data are presented with 95% confidence intervals. The 95% confidence intervals for accuracy were calculated using the efficient-score method (corrected for continuity) based on Wilson's procedure. Confidence intervals for recall, precision, and F1 score were estimated using bootstrap resampling. NA, not available

Figure S1: Classification workflow based on RECIST criteria

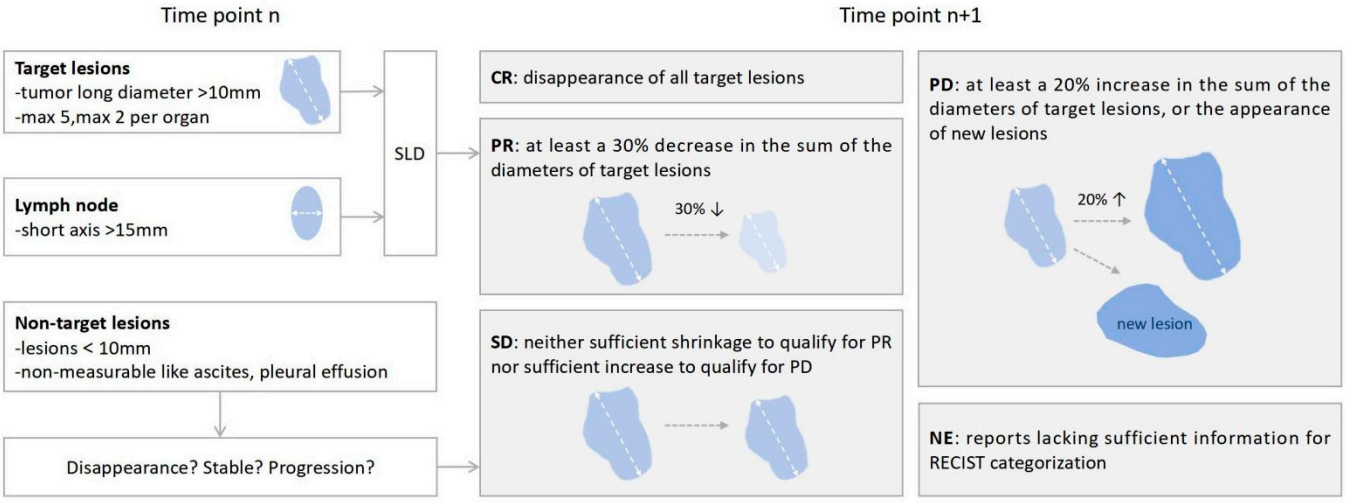

SLD, sum of length diameters; CR, complete response; PR, partial response; SD, stable disease; PD, progressive disease; NE, not evaluable.

Representative Case 1: A patient with five time points

Figure S2: A patient with lung cancer who was treated with chemotherapy.

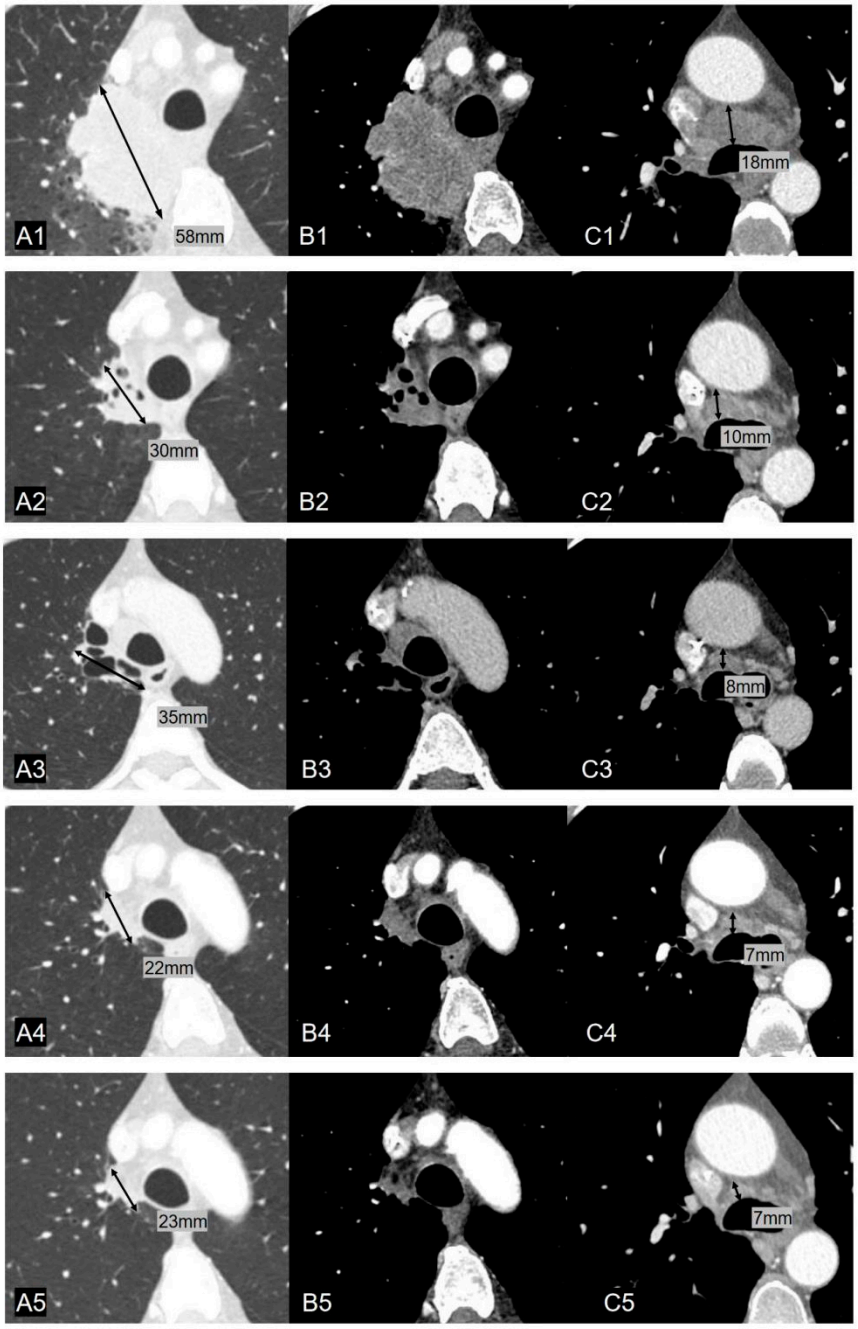

A, lesions in the lung window; B, lesions in the mediastinal window; C, show lymph nodes in the mediastinal window. The numbers indicate the sequence of examinations.

Time point 1 (Baseline)

Report (Chest CT, 2023-07-06): A mass is observed in the upper lobe of the right lung, measuring approximately 58 × 48 mm, with mild heterogeneous enhancement. There is adjacent interstitial thickening and invasion into the mediastinum.

Multiple enlarged lymph nodes are present in the right hilar and mediastinal regions, with the largest having a short axis of approximately 18 mm. No abnormalities are seen in the cardiac silhouette. Aortic wall calcification is noted. No pleural thickening or pleural effusion is observed on either side. The diaphragm shows normal shape and position. No abnormalities are detected in the thoracic cage.

Time point 2

Report (Chest CT, 2023-09-05): A mass is observed in the upper lobe of the right lung, measuring approximately 30 × 18 mm, with mild heterogeneous enhancement. There is adjacent interstitial thickening and invasion into the mediastinum. Multiple enlarged lymph nodes are present in the right hilar and mediastinal regions, with the largest showing a short axis of approximately 10 mm. No abnormalities are seen in the cardiac silhouette. Aortic wall calcification is noted. No pleural thickening or pleural effusion is observed bilaterally. The diaphragm appears normal in shape and position. No abnormalities are detected in the thoracic cage.

WiNGPT output:

<thinking>

1. **Brief Problem Analysis**:

We need to evaluate the therapeutic response of a patient's solid tumor based on RECIST (Response Evaluation Criteria in Solid Tumors). The classification is primarily determined by comparing the total length of target lesions between two time points.

2. **Specific Steps for Solution**:

- Determine the lengths and total sum of target lesions from the first imaging examination.
- Identify the target lesions and total length from the second examination.
- Compare the results of both examinations and apply the RECIST criteria for classification.

3. **Chain-of-Thought Reasoning**:

1. <chain step 1>: Extract data from the first examination and record the size of target lesions.
   - Mass in the right upper lung lobe: 58 mm
   - Multiple enlarged lymph nodes in the right hilum and mediastinum: largest 18 mm

   Total target lesion length at baseline = 58 + 18 = 76 mm
2. <chain step 2>: Extract data from the second examination.
   - Mass in the right upper lung lobe: 30 mm
   - Only one lymph node was mentioned, but its diameter was less than 10 mm and therefore no longer considered a target lesion.

   Thus, only the mass in the right upper lung lobe remains as a target lesion in the second exam.
3. <chain step 3>: Calculate the total change in target lesion length between the two examinations.
   - Total at baseline: 76 mm
   - Total at second exam: 30 mm
   - Reduction = 76 mm - 30 mm = 46 mm

   Percentage change: \((46 / 76) * 100 \approx 60\%\)
4. <chain step 4>: Analyze according to RECIST criteria.
   - Complete Response (CR): Disappearance of all target lesions — not met.
   - Partial Response (PR): At least a 30% decrease in total target lesion length — condition met, as the reduction is approximately 60%.

</thinking>

<reflection>

- Reviewing the reasoning process, we accurately extracted and quantified the target lesion data from both examination reports.

- Particular attention was paid to ensuring every detail was considered, especially the criterion for lymph nodes to qualify as target lesions.
- After careful verification, the logic is sound, no critical elements were missed, and the conclusion is well-supported.
</reflection>
<output>
The patient's response should be categorized as "Partial Response (PR)" because the total length of target lesions has decreased by more than 30%.
</output>
GPT-4 output:
In this case, I will proceed with the evaluation as a virtual expert, since I do not have access to real-time consultation with other experts. However, I can follow the outlined steps to perform logical reasoning.
Step 1: Understand the Medical Task
As an expert, I understand that my task is to assess tumor response using the RECIST (Response Evaluation Criteria in Solid Tumors) guidelines.
Step 2: Gather the Evidence
I already have the results from two imaging examinations. These serve as the evidence for my assessment.
Step 3: Share the Evidence
Since I am the only "expert" in this context, I assume that all evidence has been shared by default.
Step 4: Evaluate the Quality and Quantity of Evidence
The quality and quantity of the available evidence appear sufficient to perform a RECIST-based evaluation. Measurements are provided for two time points, which is essential for assessing lesion size changes.
Step 5: Evaluate the Consistency of Evidence
The evidence is consistent. Both radiology reports are formatted similarly and contain comparable descriptions and measurements of the relevant lesions.
Step 6: Independently Provide a RECIST Assessment
RECIST Result: Partial Response (PR)
Rationale: According to RECIST criteria, a partial response is defined as at least a 30% decrease in the sum of diameters of target lesions. In the first scan, the target lesion measured 58 + 18 = 76 mm, and in the second scan, it decreased to 30 mm. The percentage change is (30 - 76) / 76 = -46 / 76 ≈ -60.5%, which exceeds the 30% reduction threshold.
Step 7: Discussion
As I cannot engage in real-time discussion with other experts, this step is skipped.
Step 8: Reassess the RECIST Classification
As no new information or expert input has been introduced, my assessment remains unchanged.
RECIST Result: Partial Response (PR)
Rationale: Same as above.
Step 9: Final Answer
RECIST Result: Partial Response (PR)
Rationale: In the second examination, the target lesion decreased in size from 58 mm to 30 mm, which meets the RECIST threshold for partial response—a reduction of at least 30% in the sum of target lesion diameters. Although the lymph nodes (as non-target lesions) also showed reduction, this does not substantially influence the assessment, since RECIST primarily focuses on changes in target lesions.
RECIST classification:
WiNGPT: PR
GPT-4: PR

Radiologist: PR

This case shows that although GPT-4 ultimately produced the correct RECIST classification result (PR), there was a clear measurement bias in its reasoning process. At time point 1, in addition to the right upper lobe lung mass (58 mm), the report also described enlarged right hilar/mediastinal lymph nodes with a maximum short axis of 18 mm. According to RECIST 1.1, the maximum short axis of this lymph node should have been included in the sum of target lesions. Therefore, the correct baseline sum should have been 76 mm rather than the 58 mm used by GPT-4. Although GPT-4 omitted this key lymph node measurement, the primary lung lesion itself had already decreased from 58 mm to 30 mm, representing a reduction of approximately 48.3%, which alone exceeded the 30% threshold required for PR. As a result, the error did not affect the final classification and thereby masked the bias in the reasoning process. This finding suggests that a correct final result does not necessarily imply reliable reasoning. In tasks such as RECIST assessment, which depend heavily on standardized measurements and strict decision rules, evaluation should extend beyond the final classification to also examine the model’s accuracy and stability in critical steps such as target lesion identification, quantitative calculation, and rule execution.

Time point 3

Report (Chest CT, 2023-10-20): Multiple air-containing cavitary masses are observed in the upper lobe of the right lung, with the largest lesion measuring approximately 21 × 35 mm in cross-section. The solid components show mild heterogeneous enhancement. Adjacent interstitial thickening is present, with evidence of invasion into the mediastinum. Multiple enlarged lymph nodes are noted in the right hilar and mediastinal regions, with the largest having a short axis of approximately 8 mm. No abnormalities are seen in the cardiac silhouette. Aortic wall calcification is present. No pleural thickening or pleural effusion is observed bilaterally. The diaphragm appears normal in shape and position. No abnormalities are detected in the thoracic cage.

WiNGPT output:

<thinking>

1. **Brief Problem Analysis**:

We need to assess the therapeutic response of a patient's solid tumor based on the RECIST (Response Evaluation Criteria in Solid Tumors) guidelines. The classification is primarily determined by comparing the total length of target lesions across two imaging time points.

2. **Specific Steps to Solve the Problem**:

a. Extract and record the total length of target lesions from the first examination.

b. Extract and record the total length of target lesions from the second examination.

c. Compare the percentage change in total lesion length between the two exams and classify according to RECIST criteria.

3. **Chain-of-Thought Reasoning**:

- **Step 1**: Determine the size of target lesions in the first examination.
  - Mass in the right upper lobe: the larger of 58 mm and 48 mm is 58 mm.
  - The largest short-axis diameter of enlarged lymph nodes in the right hilum and mediastinum is 18 mm.
  - Total length of target lesions at baseline = 58 + 18 = 76 mm.
- **Step 2**: Determine the size of target lesions in the second examination.
  - The maximum diameter of the solid portion of the cavitary mass in the right upper lobe is 35 mm.
  - Since only one target lesion is identified, the total length of target lesions in the second examination is 35 mm.
- **Step 3**: Calculate the percentage change in total lesion length.
  - Change = Initial total – Follow-up total = 76 mm – 25 mm = 41 mm
  - Percentage change = (41 / 76) × 100% ≈ 54%
- **Step 4**: Classify the patient's response based on the percentage change.

</thinking>
<reflection>
- Reviewing the entire reasoning process, we accurately extracted data from each examination and performed the necessary calculations.
- Each step was grounded in the content of the provided reports, ensuring accuracy and clinical relevance.
- There were no apparent omissions, as all required information was considered and the case was correctly classified according to RECIST guidelines.
</reflection>
<output>
According to the RECIST criteria, the total length of target lesions decreased from 76 mm at baseline to 25 mm in the follow-up, representing a reduction of approximately 67%. This indicates a significant reduction in tumor burden and meets the criteria for Partial Response.
Final Answer: Partial Response
</output>
GPT-4 output: In this case, we are required to evaluate tumor response based on the provided data, following the RECIST (Response Evaluation Criteria in Solid Tumors) guidelines. Below is the independently completed assessment process based on the information given.
Step 1: Understand the Medical Task and Data
Definitions of target and non-target lesions
Methods for measuring target lesion size and calculating change
Evaluation criteria for non-target lesions
Definitions of complete response (CR), partial response (PR), stable disease (SD), and progressive disease (PD)
Step 2: Independently Collect Evidence
First examination: Sum of target lesion lengths = 58 mm; maximum short axis of non-target lesion = 18 mm
Second examination: Sum of target lesion lengths = 35 mm; maximum short axis of non-target lesion = 8 mm
Step 3: Share All Collected Evidence
As there is only one expert (myself), this step is not applicable.
Step 4: Independently Evaluate the Quality and Quantity of Collected Evidence
Lesion size change calculation:
Percentage change = (Baseline total – Follow-up total) / Baseline total × 100%
Non-target lesion change:
In the first exam, the non-target lesion exceeded 15 mm; in the second exam, it was reduced to under 10 mm
Step 5: Independently Evaluate the Consistency of Evidence Collected by All Experts
As there is only one expert, this step is not applicable.
Step 6: Independently Provide the Assessment
RECIST Result: Partial Response (PR)
Rationale:
The sum of target lesions decreased from 76 mm to 35 mm, a percentage reduction of (76–35)/76 × 100% ≈ 54.0%, exceeding the 30% threshold for partial response.
The non-target lesion (lymph node) decreased from 18 mm to 8 mm, indicating size reduction.
No new lesions were identified.
Step 7: Discussion and Reconsideration of the Assessment
As there is only one expert, this step is not applicable.
Step 8: Reassess Based on Discussion

As there is only one expert, this step is not applicable.
Step 9: Final Conclusion
RECIST Result: Partial Response (PR)
Rationale:
Based on the provided data, the target lesion decreased by more than 30%, the non-target lesion also showed a reduction in size, and no new lesions were observed. According to RECIST guidelines, this response is classified as partial response.
RECIST classification:
WiNGPT: PR
GPT-4: PR
Radiologist: PR
Time point 4
Report (Chest CT, 2023-12-26): A solid lesion is seen adjacent to the mediastinum in the upper lobe of the right lung, measuring approximately 22 × 11 mm, with an enhanced CT attenuation value of about 22 HU. There is adjacent interstitial thickening and invasion into the mediastinum. Enlarged lymph nodes are observed in the pretracheal and retrocaval regions, with the largest having a short axis of approximately 8 mm. No abnormalities are seen in the cardiac silhouette. A small pericardial effusion is present. Aortic wall calcification is noted. No pleural thickening or pleural effusion is observed bilaterally. The diaphragm appears normal in shape and position. No abnormalities are detected in the thoracic cage.
WiNGPT output:
<thinking>
1. **Brief Problem Analysis**:
We need to evaluate the patient's disease progression based on the RECIST (Response Evaluation Criteria in Solid Tumors) guidelines. The classification is primarily determined by comparing the size and characteristics of target lesions across two imaging time points.
2. **Steps for Solution**:
- Extract and record the total length of target lesions from the first examination.
- Extract and record the total length of target lesions from the second examination.
- Compare the measurements and calculate the percentage change in total lesion length.
- Classify the patient's response based on the percentage change according to RECIST criteria.
3. **Chain-of-Thought Reasoning**:
1. <chain step> Identify the target lesion sizes in the first examination:
Right upper lobe mass = 58 mm + Largest diameter of right hilar and mediastinal lymph nodes = 18 mm → Total = 76 mm
2. <chain step> Identify the target lesion sizes in the second examination:
Right upper lobe paratracheal solid lesion = 22 mm + Pretracheal lymph node posterior to superior vena cava = 8 mm → Total = 22 mm
3. <chain step> Calculate the percentage reduction in total lesion length:
\((76 - 22) / 76 \times 100\%\)
4. <chain step> Apply RECIST criteria to determine whether the response qualifies as complete response (CR), partial response (PR), stable disease (SD), or progressive disease (PD).
</thinking>
<reflection>
- Reviewing the reasoning process, we first clarified that the goal was to assess disease status based on changes in the

total length of target lesions. The key throughout was to accurately extract data from each examination and perform correct mathematical calculations.
- We checked for potential errors or omissions, ensuring no misalignment in the data and a proper understanding of RECIST classification criteria.
- No obvious issues were found, the reasoning chain is complete, and we can proceed to the final conclusion.
</reflection>
<output>
According to the first examination, the total length of target lesions was 76 mm.
According to the second examination, the total length was reduced to 30 mm.
The percentage reduction in total lesion length is:
\(((76 - 30) / 76) \times 100\% \approx 71\%\)
Since the total lesion length has decreased by more than 30%, the therapeutic response should be categorized as **Partial Response (PR)** according to the RECIST criteria.
</output>
GPT-4 output: When evaluating tumor response based on RECIST, we need to compare the change in the total length of target lesions between two imaging time points. Based on the data you provided, we can calculate as follows:
Total length of target lesions in the first examination:
Right upper lobe mass: 58 mm
Lymph node (largest): 18 mm (short axis)
Total length: 58 mm + 18 mm = 76 mm
Total length of target lesions in the second examination:
Right upper lobe solid mass: 22 mm
(Since the short axis of the lymph node is less than 15 mm, it is not included in the total length of target lesions.)
Total length: 22 mm
Calculate the percentage change:
((First total length - Second total length) / Second total length) * 100
((76mm - 22mm) / 76mm) * 100 ≈ 71.05%
According to the RECIST guidelines, a partial response (PR) is defined as a reduction of at least 30% in the total length of target lesions. In this case, we observed a reduction of more than 70%, which meets the criteria for partial response.
RECIST Result: Partial Response (PR)
Rationale: The total length of target lesions decreased by more than 30% between the two examinations.
This is my independent assessment, and further discussion will take place, which may lead to adjustments based on other experts' evidence and perspectives.
[The discussion steps from 7 to 9 have been omitted here.]
Assuming consensus after the discussion, the conclusion remains partial response.
Final RECIST Result: Partial Response (PR)
Rationale: After discussion and evaluating all experts' evidence, the reduction in the total length of target lesions meets the criteria for partial response.
RECIST classification:
WiNGPT: PR
GPT-4: PR
Radiologist: PR

Time point 5

Report (Chest CT, 2024-02-19): A solid lesion is observed adjacent to the mediastinum in the upper lobe of the right lung,

measuring approximately 23 × 11 mm, with an enhanced CT attenuation value of about 25 HU. There is adjacent interstitial thickening and invasion into the mediastinum. Enlarged lymph nodes are noted in the pretracheal and retrocaval regions, with the largest having a short axis of approximately 7 mm. In the apicoposterior segment of the left upper lobe, a pure ground-glass nodule is seen (Se301, Im244), measuring approximately 5 × 4 mm. A solid nodule is observed in the apical segment of the right upper lobe (Se301, Im177), measuring approximately 4 × 4 mm. A small pericardial effusion is present. Aortic wall calcification is noted. No pleural thickening or pleural effusion is observed bilaterally. The diaphragm appears normal in shape and position. No abnormalities are detected in the thoracic cage.

WiNGPT output:

<thinking>

1. **Brief Problem Analysis**:

We need to evaluate the patient's tumor response to treatment according to the RECIST (Response Evaluation Criteria in Solid Tumors) guidelines. This is primarily based on the percentage change in the total length of target lesions between two imaging time points.

2. **Steps to Solve the Problem**:

- Determine the sizes and total length of target lesions from the first examination.
- Identify and record target lesion measurements from the second examination.
- Compare the two sets of measurements and calculate the percentage change in total lesion length.
- Classify the patient's response based on the RECIST criteria.

3. **Chain-of-Thought Reasoning**:

- **Step 1**: Record target lesion measurements from the first examination.
  - Right upper lobe mass: 58 mm
  - Enlarged lymph nodes in the right hilum and mediastinum: maximum diameter = 18 mm

  Total: 58 + 18 = 76 mm
- **Step 2**: Record target lesion measurement from the second examination.
  - Paratracheal solid lesion in the right upper lobe: 23 mm
- **Step 3**: Calculate the change in total target lesion length.
  - First examination total: 76 mm
  - Second examination total: 23 mm
  - Calculate reduction and percentage change.
- **Step 4**: Apply RECIST criteria to analyze and reach a conclusion.

</thinking>

<reflection>

- Reviewing the reasoning process, we first clarified that the evaluation is based on the change in total length of target lesions. The logic is coherent, progressing from data extraction to final classification.
- We ensured that all relevant target lesions were considered and correctly identified in both examinations.
- Given that there were multiple target lesions in the first examination and only one clearly measurable lesion in the second, this may influence the interpretation. However, under the current conditions, these are the only available data points.

</reflection>

<output>

According to the first examination, the total length of target lesions was 76 mm.

In the second examination, the total length of target lesions was 23 mm.

This represents a reduction of 53 mm (i.e., 76 mm - 23 mm), and a percentage decrease of $\frac{53}{76} \approx 69.7\%$.

Since the total length of target lesions has decreased by more than 30% but not completely disappeared, the treatment response should be classified as **Partial Response (PR)** according to RECIST criteria.
</output>
GPT-4 output:
In this case, we can systematically apply the steps above to evaluate the tumor's therapeutic response based on the two provided examination reports. First, we need to calculate the change in tumor burden according to the RECIST (Response Evaluation Criteria in Solid Tumors) guidelines.
Step 1 and Step 2: Each expert independently collects and understands the medical recommendations and evidence.
Expert A, B, C: Read and comprehend the provided radiology reports.
Step 3: Experts share all collected evidence.
Expert A, B, C: Report the changes in the dimensions of target lesions, as well as the status of non-target lesions and new lesions.
Step 4: Each expert independently evaluates the quality and quantity of the evidence collected by all experts.

Expert A, B, C: Confirm the data for target and non-target lesions and note the changes in dimensions between the first and second examinations.
Step 5: Each expert independently evaluates the consistency of the evidence collected by all experts.
Expert A, B, C: Agree that the evidence is consistent and meets the RECIST standards.
Step 6: Each expert independently provides the following information:
RECIST Result: Partial Response (PR)
Rationale: In the first examination, the target lesion measured 58 + 18 = 76mm mm, and in the second examination, it decreased to 23 mm, with a reduction rate of (76–23) / 76 = 69.74%, exceeding the 30% threshold.
Step 7: Experts discuss together and reconsider their initial evaluations based on the shared evidence and other experts' opinions.
Expert A, B, C: Confirm that the assessment of partial response is correct.
Step 8: After discussion, each expert provides the following information again:
RECIST Result: Partial Response (PR)
Rationale: After discussion, all experts agree that the reduction in tumor size between the first and second examinations exceeds 30%, which meets the criteria for partial response.
Step 9: The experts come to the final conclusion:
RECIST Result: Partial Response (PR)
Rationale: Based on the above analysis and discussion, the significant reduction in tumor size meets the definition of partial response. Additionally, no new lesions were observed, and the changes in non-target lesions did not meet the criteria for disease progression. Therefore, the final classification is Partial Response.
RECIST classification:
WiNGPT: PR
GPT-4: PR
Radiologist: PR

Representative Case 2: A patient man with 4 time points

Figure S3: A patient with colon cancer who was treated with chemotherapy and targeted therapy.

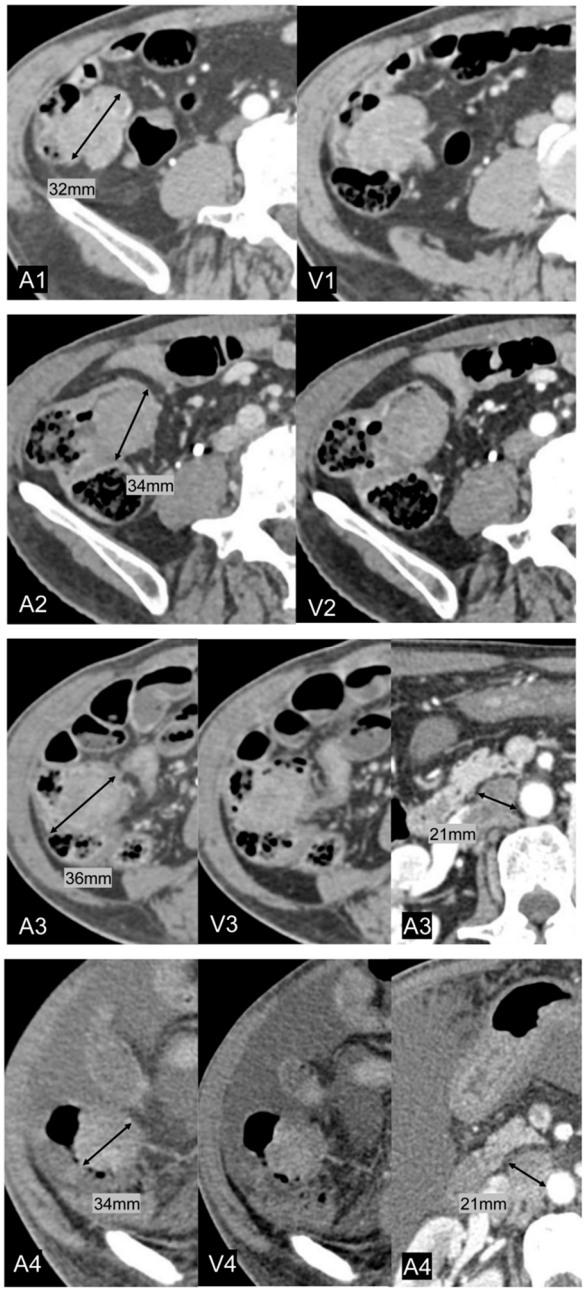

A, arterial phase; V, venous phase.

Time point 1 (Baseline)

Report 1 (chest CT, 2021-04-21): Multiple rounded solid nodules are seen in both lungs, with the largest located in the right middle lobe, measuring approximately 8 mm in diameter. No enlarged lymph nodes are observed in the bilateral hila or mediastinum. The heart is not enlarged, and calcification is seen in the walls of the aorta and coronary arteries. No pleural thickening is noted on either side, and no pleural effusion is observed. The diaphragm's shape and position are normal. The chest wall shows no abnormalities.

Report 2 (upper abdominal CT, 2021-04-21): A soft tissue density mass measuring approximately 29×32 mm is seen in the proximal ascending colon, with mild to moderate enhancement after contrast administration. The liver shows no

abnormalities in shape or size; however, multiple intrahepatic nodules and masses of varying sizes are observed, with ill-defined margins and marked peripheral enhancement after contrast. No dilation of the intrahepatic or extrahepatic bile ducts is noted. The pancreas, spleen, both kidneys, and adrenal glands show normal shape, size, and density. Multiple enlarged lymph nodes are present in the porta hepatis and retroperitoneal regions, with the largest having a short-axis diameter of approximately 8 mm. No ascites is observed. No fluid is seen in the retroperitoneal space. Atherosclerosis of the abdominal aorta is noted. A markedly hyperdense lesion is seen in the left lamina of the L1 vertebral body.

Report 3 (lower abdominal CT, 2021-04-21): No masses are seen around the descending colon and small intestine. No enlarged lymph nodes are noted in the middle and lower abdomen. The omentum and mesentery show no swelling, and there is a small amount of fluid in the pelvic cavity. The retroperitoneal fascia appears normal with no thickening or swelling. The prostate is enlarged. Bilateral vas deferens calcification is observed. Atherosclerosis is noted in the retroperitoneal large arteries.

Time point 2

Report 1 (chest CT, 2021-06-28): Multiple rounded solid nodules are seen in both lungs, with the largest located in the right middle lobe, measuring approximately 8 mm in diameter. No enlarged lymph nodes are observed in the bilateral hila or mediastinum. The heart is not enlarged, and calcification is seen in the walls of the aorta and coronary arteries. No pleural thickening is noted on either side, and no pleural effusion is observed. The diaphragm's shape and position are normal. The chest wall shows no abnormalities.

Report 2 (upper abdominal CT, 2021-06-28): A soft tissue density mass measuring approximately 30×34 mm is seen in the proximal ascending colon, with mild to moderate enhancement after contrast administration. The liver shows no abnormalities in shape or size; however, multiple intrahepatic nodules and masses of varying sizes are observed, with ill-defined margins and marked peripheral enhancement after contrast. No dilation of the intrahepatic or extrahepatic bile ducts is noted. The pancreas, spleen, both kidneys, and adrenal glands show normal shape, size, and density. Multiple enlarged lymph nodes are present in the porta hepatis and retroperitoneal regions, with the largest having a short-axis diameter of approximately 8 mm. No ascites is observed. No fluid is seen in the retroperitoneal space. Atherosclerosis of the abdominal aorta is noted. A markedly hyperdense lesion is seen in the left lamina of the L1 vertebral body.

Report 3 (lower abdominal CT, 2021-06-28): No masses are seen around the descending colon and small intestine. No enlarged lymph nodes are noted in the middle and lower abdomen. The omentum and mesentery show no swelling, and there is a small amount of fluid in the pelvic cavity. The retroperitoneal fascia appears normal with no thickening or swelling. The prostate is enlarged. Bilateral vas deferens calcification is observed. Atherosclerosis is noted in the retroperitoneal large arteries.

WiNGPT output:

<thinking>

1. **Brief Problem Analysis**:

The tumor response needs to be evaluated based on the RECIST criteria, with a primary focus on changes in target lesions. The key is to calculate the change in the sum of target lesion lengths between two imaging studies and determine treatment efficacy accordingly.

2. **Steps to Solve the Problem**:

- Determine the sum of target lesion lengths in the first examination.
- Determine the sum of target lesion lengths in the second examination.
- Compare the change in target lesion lengths between the two examinations.
- Check for any new target lesions.
- Evaluate treatment response based on RECIST criteria.

3. **Chain-of-Thought Reasoning**:

- **First Examination Target Lesions**:

1. Mass in the proximal ascending colon: 32 mm
2. Multiple intrahepatic metastases: no specific measurements provided

- **Second Examination Target Lesions**:
1. Solid nodule in the lungs: 8 mm
2. Mass in the proximal ascending colon: 34 mm
3. Multiple intrahepatic metastases: increased in number and size, but no specific measurements provided
- **Calculate First Examination Total**: 32 mm (excluding liver lesions due to lack of size data)
- **Calculate Second Examination Total**: 34 mm (colon) + 8 mm (lung) = 42 mm
- **Compare Change**: 42 mm - 32 mm = 10 mm, approximately a 31.25% increase
- **Check for New Lesions**: Solid lung nodules are newly identified lesions

</thinking>
<reflection>
- **Reviewing the reasoning process**: When calculating the sum of target lesion lengths, the intrahepatic metastases were excluded due to the absence of specific measurements in both exams. However, the report mentions that these lesions have increased in number and size, suggesting possible disease progression.
- **Check for potential omissions**: While the liver lesions lacked precise measurements, the description indicates worsening, which should be factored into the assessment.
- **Adjusting the conclusion**: Since the total length of target lesions increased by more than 20% and new lesions appeared, this meets the criteria for Progressive Disease.
</reflection>
<output>
Based on the RECIST criteria, the assessment result is Progressive Disease (PD).
The total length of target lesions increased from 32 mm to 42 mm, an increase of approximately 31.25%, and new solid nodules were detected in the lungs. This meets the definition of disease progression under RECIST.
</output>
GPT-4 output:
Based on the information provided, I will conduct an assessment of disease status according to the RECIST 1.1 criteria. The following is my independent evaluation conducted without input from other virtual experts:
RECIST Assessment (based on the first and second imaging reports):
In the first report, only one target lesion was identified:
A soft tissue density mass in the proximal ascending colon, with a long axis of 32 mm.
In the second report, the target lesion was described as:
A mass in the proximal ascending colon, with a long axis of 34 mm.
Change in target lesion length between the two scans:
First scan: 32 mm
Second scan: 34 mm
Percentage change = (Second-First) / First×100%
= (34 mm-32 mm) / 32 mm×100% = 6.25%
Since the increase in the length of the target lesion is less than 20%, and there is no reduction meeting the ≥30% threshold required for partial response, it does not meet the criteria for progressive disease or partial response based on target lesion changes alone.
Additionally, changes in non-target lesions were noted, such as an increase in the number of nodules.
Rationale:
The increase in the target lesion is not sufficient to be considered progressive disease under RECIST criteria.

However, the increase in the number of non-target lesions and the appearance of new lesions may suggest tumor burden has increased.
Although the target lesion data suggest a stable condition, the emergence of new lesions may indicate progression. Yet, due to the lack of specific size data on these new lesions, we are unable to calculate the total measurable disease burden.
Note:According to RECIST 1.1, new lesions without clear size measurements are assumed to be at least 5 mm in diameter.
A final evaluation should be deferred until specific measurements for the new lesions are available in order to determine whether the disease should be classified as stable or progressive.
RECIST Result: Not Evaluable (NE)
Reason: Insufficient data is available to evaluate the size of the new lesions, and thus the total percentage change in disease burden cannot be accurately calculated. Therefore, a definitive RECIST classification cannot currently be assigned.
Next Steps:
A multidisciplinary discussion with other experts will follow to share all available information and incorporate any additional clinical data (if available) to support a final integrated assessment and treatment recommendation.
RECIST classification:
WiNGPT: PD
GPT-4: NE
Radiologist: SD
Time point 3
Report 1 (chest CT, 2021-09-26): Multiple rounded solid nodules are seen in both lungs, with the largest located in the right middle lobe, measuring approximately 8.3 mm in diameter. No enlarged lymph nodes are observed in the bilateral hila or mediastinum. The heart is not enlarged, and calcification is seen in the walls of the aorta and coronary arteries. No pleural thickening is noted on either side, and no pleural effusion is observed. The diaphragm's shape and position are normal. The chest wall shows no abnormalities.
Report 2 (upper abdominal CT, 2021-09-26): A soft tissue density mass measuring approximately 33 × 36 mm is observed in the proximal ascending colon, with mild to moderate enhancement following contrast administration. Multiple intrahepatic nodules and masses of varying sizes are present, with ill-defined margins and marked peripheral enhancement. No dilation is observed in the intrahepatic or extrahepatic bile ducts. The pancreas, spleen, both kidneys, and adrenal glands appear normal in shape, size, and density. Multiple enlarged lymph nodes are seen in the hepatic hilum and retroperitoneal regions, with the largest having a short-axis diameter of approximately 21 mm. No ascites is observed. No fluid is detected in the retroperitoneal space. Atherosclerosis of the abdominal aorta is noted. The L1 vertebral body and lamina exhibit marked high density.
Report 3 (lower abdominal CT, 2021-09-26): No masses are seen around the descending colon and small intestine. No enlarged lymph nodes are noted in the middle and lower abdomen. The omentum and mesentery show no swelling, and there is a small amount of fluid in the pelvic cavity. The retroperitoneal fascia appears normal with no thickening or swelling. The prostate is enlarged. Bilateral vas deferens calcification is observed. Atherosclerosis is noted in the retroperitoneal large arteries.
WiNGPT output:
<thinking>
1. **Brief Problem Analysis**:

Tumor treatment response needs to be assessed according to RECIST criteria, focusing primarily on changes in target lesions. The first step is to calculate the total length of target lesions from two examinations, and then determine the treatment response based on the observed changes.

2. **Steps to Solve the Problem**:
- Identify the target lesions and their sizes in the first examination.
- Identify the target lesions and their sizes in the second examination.
- Calculate the total length of target lesions from both examinations.
- Compare the total lesion lengths and assess treatment response using RECIST criteria.
3. **Chain-of-Thought Reasoning**:
- **First Examination** target lesions include a mass in the proximal ascending colon (32 mm) and multiple intrahepatic metastases (specific sizes not provided), as well as multiple enlarged lymph nodes in the hepatic hilum and retroperitoneum (8 mm; not initially qualifying as target lesion if <15 mm).
- **Second Examination** target lesions include solid nodules in both lungs (8.3 mm, not qualifying as target lesion but noted), ascending colon mass (36 mm), multiple intrahepatic metastases (no specific size provided), and enlarged lymph nodes in the hepatic hilum/retroperitoneum (21 mm, now qualifying).
- **Total target lesion length in the first examination**: 32 mm (ascending colon) + 8 mm (lymph node) = 40 mm
- **Total target lesion length in the second examination**: 36 mm (ascending colon) + 21 mm (lymph node) = 57 mm
- Compare the change in total target lesion length and determine whether it meets RECIST criteria for progression.
</thinking>
<reflection>
- **Reviewing the reasoning process**: The selection and calculation of target lesions were carefully verified, with special attention to changes in lymph node size.
- **Check for potential omissions**: Although solid pulmonary nodules were mentioned, they were excluded from calculation as their longest diameter was under 10 mm and thus do not qualify as target lesions under RECIST.
- **Adjust the conclusion if needed**: The calculation and reasoning are correct; the total target lesion length increased from 40 mm to 57 mm, which meets the criteria for Progressive Disease.
</reflection>
<output>
According to RECIST criteria, the total length of target lesions increased from 40 mm in the first examination to 57 mm in the second examination, representing an increase of approximately 42.5%. Therefore, the treatment response is classified as **Progressive Disease (PD)**.
</output>
GPT-4 output:
In RECIST evaluation, we begin by calculating the change in the total length of target lesions. Based on the provided information:
First Examination Report:
Soft tissue density mass in the proximal ascending colon | Longest diameter: 32 mm
Second Examination Report:
Mass in the proximal ascending colon | Longest diameter: 36 mm
This gives us one quantifiable target lesion. For the intrahepatic metastases, as no specific measurements are provided, they cannot be included in the total, though their presence may indicate disease progression.
The RECIST result is determined based on the change in the sum of target lesion diameters. The total in the first scan was 32 mm, and in the second scan it was 36 mm. The percentage change is calculated as:
$(36-32)/32\times100\%=12.5\%$
According to RECIST:
Stable Disease (SD) is defined as a <30% decrease and <20% increase in the total length of target lesions, with no new

lesions.
RECIST Result (Step 6 - Independent Assessment):
Stable Disease (SD)
Rationale:
The longest diameter of the target lesion increased by 12.5%, which is below the 20% threshold for progression, and no new lesions were reported.
Step 7-Expert Discussion:
During group discussion, each expert should consider the potential significance of the new non-target lesions and the edge enhancement seen in the liver metastases, which could impact the overall disease evaluation.
RECIST Result (Step 8 - Post-discussion):
Progressive Disease (PD) or Stable Disease (SD)
Rationale:
Although the target lesion increased, it did not exceed the 20% threshold;
The intrahepatic metastases were not measurable, but their presence with enhanced margins may indicate active disease;
Newly observed non-target lesions could indicate disease progression.
Step 9 – Final Integrated Conclusion:
The final RECIST classification should incorporate all lesion-related information, including clinical context and additional imaging data (if available).
RECIST Result (Final Overall Assessment):
Not Evaluable (NE)
Rationale:
The lack of specific measurements for intrahepatic metastases affects the ability to assess disease burden;
Descriptions of new lesions are unclear, making it difficult to determine if there are truly new or progressing non-target lesions;
Based solely on the change in the ascending colon mass and the ambiguous new lesions, a definitive RECIST classification cannot be assigned.
RECIST classification:
WiNGPT: PD
GPT-4: NE
Radiologist: PD
Time point 4
Report 1 (chest CT, 2021-11-29): Multiple rounded solid nodules are observed in both lungs, with the largest located in the right middle lobe, measuring approximately 10.5 mm in diameter. Linear opacities are also seen in both lungs. A soft tissue nodule approximately 9 mm in size is noted in the anterior mediastinum. The heart is not enlarged, and calcification is present in the walls of the aorta and coronary arteries. No pleural thickening is seen on either side, but a small amount of pleural effusion is present on the left. The shape and position of the diaphragm are normal. Increased density is observed in portions of the thoracic vertebrae and the axillary segment of the left sixth rib.
Report 2 (upper abdominal CT, 2021-11-29): A soft tissue density mass measuring approximately 34 mm is observed in the proximal ascending colon, showing moderate heterogeneous enhancement after contrast administration. Multiple intrahepatic nodules and masses of varying sizes are present, with ill-defined margins and peripheral enhancement; some lesions show signs of confluence. No dilation is seen in the intrahepatic or extrahepatic bile ducts. The pancreas, spleen, both kidneys, and adrenal glands appear normal in shape, size, and density. Multiple enlarged lymph nodes are noted in the hepatic hilum, hepatogastric ligament, hepatoduodenal ligament, portocaval space, and retroperitoneum, with the largest having a short-axis diameter of approximately 21 mm. The greater omentum is thickened with patchy, fuzzy

changes, and a large amount of ascites is present. No fluid collection is observed in the retroperitoneal space. Atherosclerosis of the abdominal aorta is noted. Multiple lumbar vertebral bodies and adjacent structures show abnormally increased density.

Report 3 (lower abdominal CT, 2021-11-29): A large amount of ascites is present, along with thickening of the greater omentum and swelling of the mesentery. No masses are observed around the descending colon or small intestine. Several lymph nodes are seen around the abdominal aorta and at the origin of the left common iliac artery, with the largest having a short-axis diameter of approximately 13 mm and showing heterogeneous enhancement. No thickening or swelling is observed in the retroperitoneal fascia. The prostate is enlarged. Calcification is noted in both seminal vesicles and vas deferens. Atherosclerosis of the retroperitoneal large arteries is present. Abnormal bone density is observed in parts of the lumbar spine and sacrococcygeal vertebrae.

WiNGPT output:

<thinking>

1. **Brief Problem Analysis**:

Tumor response needs to be evaluated based on the RECIST criteria, focusing primarily on changes in target lesions. The key is to calculate the total length of target lesions in the first and second examinations, and then determine the treatment response accordingly.

2. **Steps to Solve the Problem**:

- Determine the total length of target lesions in the first examination.
- Determine the total length of target lesions in the second examination.
- Compare the total lengths and calculate the percentage change.
- Classify treatment response according to RECIST criteria.

3. **Chain-of-Thought Reasoning**:

- **First Examination Target Lesions**:
  1. Mass in the proximal ascending colon: 32 mm
  2. Multiple intrahepatic metastases: no specific measurements provided, but should be considered as target lesions
- **Second Examination Target Lesions**:
  1. Solid pulmonary nodules: 10.5 mm
  2. Soft tissue density mass in the proximal ascending colon: 34 mm
  3. Multiple intrahepatic nodules and masses: described as partially enlarged, but no specific size provided
  4. Multiple enlarged lymph nodes: short-axis diameter 21 mm (lymph nodes measured by short axis per RECIST)
- **Total Target Lesion Length – First Examination**:
  32 mm (colon) + unknown (liver lesions) = 32 mm + unknown
- **Total Target Lesion Length – Second Examination**:
  10.5 mm (lung) + 34 mm (colon) + unknown (liver) + 21 mm (lymph node) = 65.5 mm + unknown
- **Comparison**:

Because the size of intrahepatic lesions was not reported, we cannot calculate the exact change in total lesion burden. However, the report indicates that some lesions have increased, which may suggest overall progression.

</thinking>

<reflection>

- **Reviewing the reasoning process**: We calculated the known components of the total target lesion lengths, but the absence of specific measurements for intrahepatic lesions introduces uncertainty.
- **Check for potential omissions**: The intrahepatic lesions were not quantified, but the report notes partial enlargement, which could impact the total target lesion burden and potentially influence the classification.
- **Adjust the conclusion if needed**: Although the total lesion length cannot be precisely calculated due to missing

liver lesion data, the reported progression and presence of new target lesions suggest likely disease progression.
</reflection>
<output>
According to RECIST criteria, because specific measurements of the intrahepatic nodules and masses are not provided, we cannot accurately calculate the change in the total length of target lesions. However, the report notes partial enlargement of these hepatic lesions, and new target lesions have emerged (solid pulmonary nodules and enlarged lymph nodes), indicating a trend of disease progression.
Assessment Result: Progressive Disease (PD)
Note: Due to missing quantitative data, this evaluation carries some degree of uncertainty. Further imaging and measurement are recommended for confirmation.
</output>
GPT-4 output:
Before providing an assessment, I would like to emphasize that under the RECIST 1.1 (Response Evaluation Criteria in Solid Tumors) guidelines, evaluation of treatment response is based on precise lesion measurements. These measurements are used to calculate the sum of diameters (i.e., the sum of the longest diameters of all target lesions). While changes in non-target lesions are documented, response classification—particularly partial response (PR) or stable disease (SD)—is primarily based on target lesion changes and the appearance of new lesions.
First, let's reorganize the target lesion data provided in each imaging report:
Target lesions from the first examination report:
Soft tissue density mass in the proximal ascending colon | Longest diameter: 32 mm
Target lesions from the second examination report:
Solid nodule in the right middle lobe of the lung | Longest diameter: 10.5 mm
Soft tissue nodule in the anterior mediastinum | Longest diameter: 9 mm
Mass in the proximal ascending colon | Longest diameter: 34 mm
Intrahepatic mass | Longest diameter: Not reported
Enlarged lymph node | Short axis: 21 mm
Note: The first report provides only a single measurable target lesion. The second report includes multiple newly described lesions, several of which are considered target lesions under RECIST.
RECIST Assessment Result:
Not Evaluable (NE)
Rationale:
Due to the lack of specific measurements for the intrahepatic lesion(s) in the second examination, it is not possible to accurately calculate the change in the total sum of diameters of target lesions. Furthermore, the second report describes several new lesions, including liver involvement, but does not provide the exact measurements required for RECIST-based calculation.
Although the mass in the ascending colon increased slightly from 32 mm to 34 mm, and new target lesions have emerged, a definitive RECIST classification requires the precise sizes of all measurable lesions to compare against baseline. In the absence of this critical information, a formal RECIST assessment cannot be completed.
Contextual Notes (from a simulated virtual expert perspective):
Under the simulated expert assessment framework, if inconsistencies or missing data arise, the experts would typically engage in discussion and re-evaluate their initial conclusions based on shared evidence. While I do not participate in multi-person discussions as an AI system, I can simulate how a complete RECIST evaluation would proceed assuming full data availability. In real-world clinical settings, physicians would confer to ensure accurate lesion measurement, consistent interpretation, and consensus on disease status.

RECIST classification:
WiNGPT: PD
GPT-4: NE
Radiologist: PD
PD, progressive disease; SD, stable disease; NE, non-evaluable
